# Practice variation in home care nursing: mapping potential explanations through a scoping review of the literature

**DOI:** 10.1007/s43999-024-00048-8

**Published:** 2024-08-21

**Authors:** A.E.M. Brabers, M.A.M. Meijer, P. P. Groenewegen, N. Bleijenberg, S. Zwakhalen, J.D. de Jong

**Affiliations:** 1 Nivel – Netherlands Institute for Health Services Research, Utrecht, The Netherlands; 2 Department of Sociology and Department of Human Geography, Utrecht University, Utrecht, The Netherlands; 3 University of Applied Sciences Utrecht, Utrecht, The Netherlands; 4 University Medical Center Utrecht, Utrecht, The Netherlands; 5 Care and Public Health Research Institute, Department of Health Services Research, Maastricht University, Maastricht, The Netherlands; 6 Living Lab in Ageing and Long Term Care, Maastricht, The Netherlands; 7 Care and Public Health Research Institute, Department of Health Services Research, Maastricht University, Maastricht, The Netherlands

**Keywords:** Practice variation, Home care, Needs assessment, Nursing

## Abstract

Needs assessment is the starting point of good home care as it determines which care is necessary, based on the needs of patients, their personal situation, and social context. There are indications that practice variation in needs assessment exists among home care nurses. However, little is known about potential explanations for this variation. Therefore, we explored potential explanations for practice variation in other areas and examined whether these explanations can be applied to explain variation in needs assessment in home care nursing. We conducted a scoping review of the literature on practice variation in (1) needs assessment in home care nursing, (2) home care nursing in general, and (3) medical care in general, with searches in PubMed and CINAHL. We assessed over 6,000 references. Ultimately, 386 studies were included. Explanations for practice variation were grouped into micro, meso and macro level. This scoping review provided insight into a wide variety of variables that might play a role in explaining practice variation in (needs assessment in) home care nursing, such as availability of guidelines, organisational culture, team norms, resources, and preferences of patients. However, the small literature on needs assessment by home care nurses devoted more attention to patients and their social context, compared to the literature on practice variation in general. We discuss how and to what extent these variables could relate to practice variation in (needs assessment in) home care nursing. Future research should empirically examine the role of these variables in explaining the observed practice variation.

**Supplementary Information:**

The online version contains supplementary material available at 10.1007/s43999-024-00048-8.

## Introduction

This paper started out to investigate what is known about potential explanations for practice variation in needs assessment in home care nursing, which is the case when patients with similar care needs, personal situation and social context are assessed to receive different types or different amounts of care [1]. We performed a scoping review of the published literature. We started with a review of the literature on needs assessment in home care. Needs assessment is the starting point of good home care provision as it determines which care is necessary based on the care needs of patients, their personal situation, and social context [2]. It turned out that there is not much literature on practice variation in needs assessment. We therefore moved our focus to practice variation in home care nursing in general. Finding a low number of studies about practice variation in home care nursing, we finally moved to practice variation in general in order to gain insight into explanations for practice variation in general, and to examine whether these can be applied to home care nursing, and more specifically to practice variation in needs assessment. In this article we follow these steps of our research.

Home care nursing is an important and relevant subject in applied health services research. There is an increased reliance on home care nursing in European countries due to growing demands for care and shift of care towards the community. The main reasons for this shift are the assumed lower costs, compared to care in institutions, and the preferences of people in need of care to live as long as possible in their own home in the community [3]. With the increased reliance on home care nursing [3], practice variation in home care nursing is becoming more important for policy-makers. Practice variation can be warranted and unwarranted and may point to under- or over-utilisation of care. Our study was motivated by policy changes in Dutch long-term care in 2015 (see Box 1).


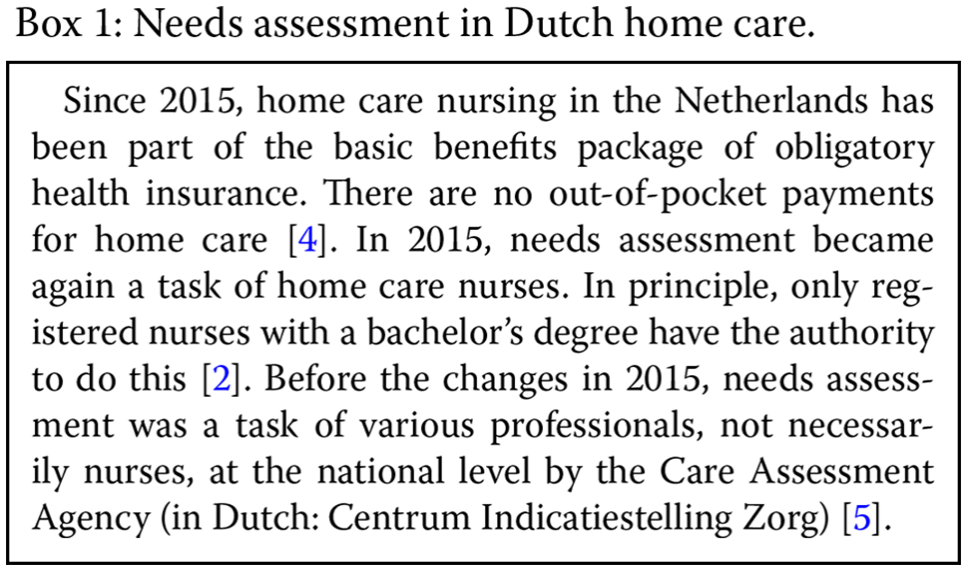


Specific for variation in needs assessment in home care nursing, a definition was developed in a Delphi study: “Variation in needs assessment is the way the home care nurses differ in the nature, amount and duration of care they indicate for clients in similar situations” [5]. People with comparable care needs, personal situation and social context should receive the same type, amount and duration of care. The cited definition is a specification of general definitions of practice variation, e.g. “the degree to which healthcare providers differ in the frequency and/or way in which care is provided to patients with comparable care problems” [6], and: “variation that cannot be explained on the basis of illness, medical evidence or patient preferences” [1]. Hence, variation that is related to clinically relevant differences between patients or different care problems, does not fall under the commonly used definitions of practice variation. The underlying causes of practice variation are often unclear, because care providers are mostly not able to clarify why variation exists. Policy-makers and funders often see variation as a signal of unnecessary care [7].

There is currently a lack of knowledge about practice variation in needs assessment in home care nursing; however, there is anecdotal evidence from the Dutch assessment practice, and some research, pointing to the existence of practice variation in needs assessments performed by Dutch home care nurses. For instance, Buijs et al. [8] showed practice variation in needs assessment in home care in their study in which care intakers assessed vignettes. Also a more recent study [9] indicated practice variation in home care nursing. The authors found that for one specific vignette the amount of hours for nursing care assessed by nurses varied from 4 to 13 h per week. The culture of the organisation in which nurses work and usual practice within the organisation was mentioned in this study as a potential explanation for the observed variation, but this hypothesis was not tested [9]. Also in other countries practice variation in the assessment and provision of home care has been observed [10–12]. The study of Van Hout et al. [12] showed substantial variations in provided formal care time among 33 organizations both within and across six European countries. These variations could not be explained by the case-mix differences of patients [12]. The existence of practice variation in needs assessment can imply that part of the care is ineffective: some patients are offered too much care, while others are offered too little. However, insight into the extent of the variation, both in types and amounts of care, and the underlying causes is lacking [13].

**Fig. 1:**
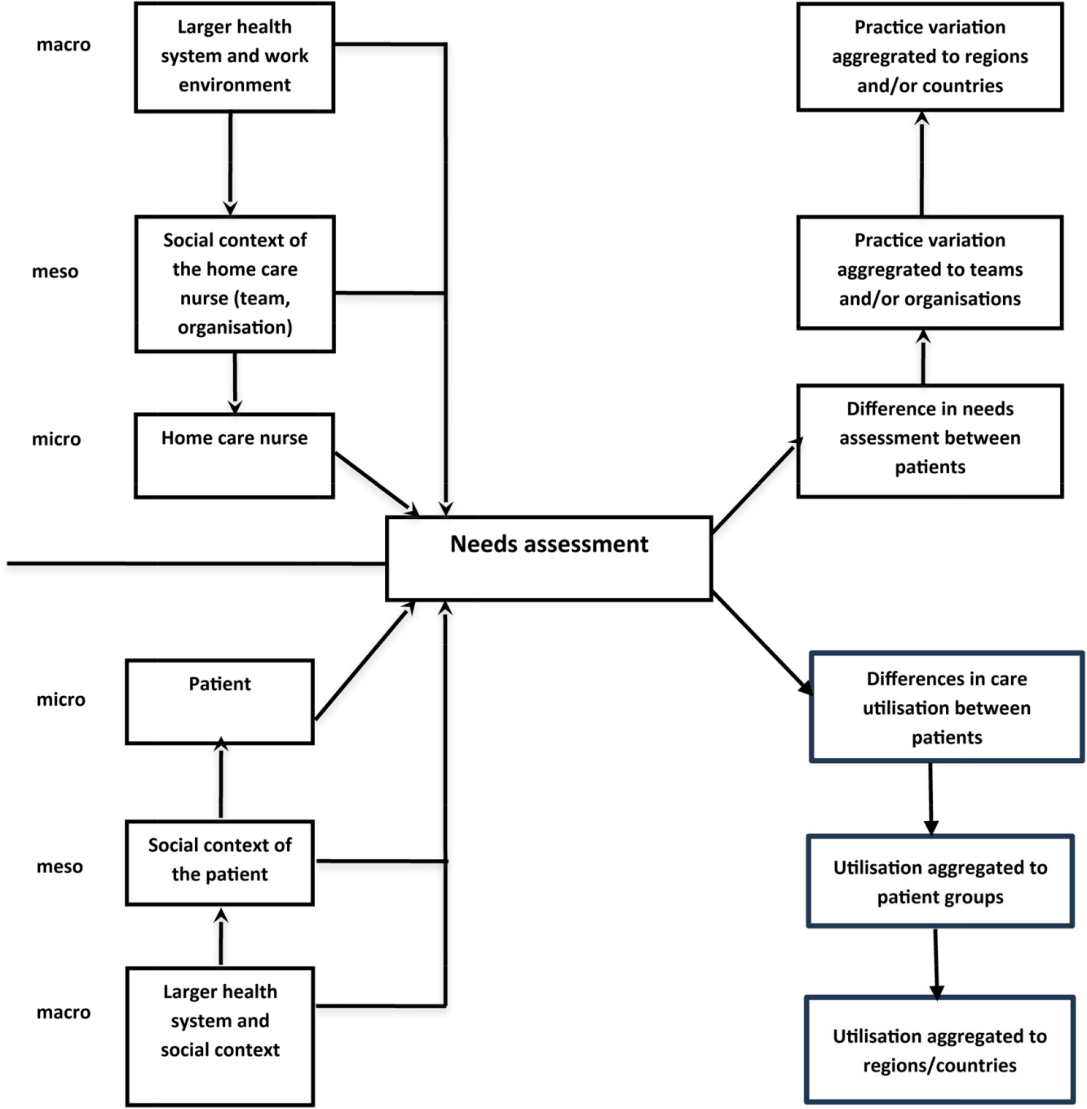
Theoretical model (based on [14, 15])

The underlying causes of variation in needs assessment may be located at different levels and both at the provider and patient side, as illustrated in Fig. 1 [adapted from: 14, 15]. At the provider side, we distinguish between the micro level of individual professionals, the meso level of the organisations and teams the professionals work in and the macro level of the health system. At each of these levels there may be practice variation. At the patient side the same levels are relevant (the micro level of the patient, the meso level of patients in social groups and the macro level again of the health system) and at each of these level there is variation in utilisation of care. The level at which variation is found informs about where explanations for the occurrence of variation have to be sought. In the case of home care nursing, the amounts and types of care may depend on characteristics of the patients and the individual home-care nurses, the teams in which the home-care nurses works, and the organisations they work at. A review of the literature may give clues as to the relevance of these levels and the specific influences at each of these levels.

We performed a scoping review to fill the gap in insight in the causes of practice variation in needs assessment in home care. Our initial objective was to gain insight into explanations for practice variation in needs assessment in home care nursing. There was only a small amount of published research in this area. We could have stopped here, but that would not have filled the knowledge gap. We decided instead to broaden up our review, first to include practice variation in home care nursing and secondly to include practice variation in general. We studied the literature on practice variation in general to gain an overview of potential explanations and to examine whether these explanations can be applied to home care nursing, and more specifically to practice variation in needs assessment.

## Methods

### Design

We performed a scoping review [16] of the literature on practice variation in home care nursing and broader in medical care in general. We conducted a scoping review because the literature on the subject is very heterogenous [17]. As we were looking for potential explanations at the micro, meso and macro level for variation in home care nursing practice, a systematic review of quantitative information is not possible nor will lead to the information we are looking for. The approach is rather a mapping exercise of information from research into potential explanations of variation, in order to generate insights that may be tested in future studies on variation in home care nursing. In performing the scoping review, we broadly followed the checklist PRISMAExtension for Scoping Reviews (PRISMA-ScR) (Appendix C).

### Search strategies

At the start of the review process and based on previous searches of the literature, we anticipated that there would not be much literature on variation in needs assessment in home care nursing, and perhaps also not on practice variation in home care nursing. We therefore performed three different strategies in PubMed and/or CINAHL (Cumulative Index to Nursing and Allied Health Literature): (1) a specific search on practice variation in needs assessment in home care nursing, conducted in PubMed on June 5, 2023 (73 articles) and in CINAHL on June 19, 2023 (152 articles); (2) a general search on practice variation in home care nursing, conducted in PubMed (673 articles) and in CINAHL, both on June 19, 2023 (535 articles), and (3) a search into medical practice variation in general (i.e. all care necessary for a person’s health and well-being provided by a doctor, nurse, or other healthcare professional), conducted in PubMed only on June 19, 2023 (5,098 articles). For the first search we divided the search strategy into three blocks, the first block was about ‘needs assessment’, the second about ‘home care nursing’, and the third about ‘variation’. For the second search we combined the second and third block. For the third strategie, we only focused on specific search terms regarding variation, like ‘practice pattern’ and ‘small-area analysis’. Our main focus was on practice variation and thus on the role of care providers. Variation in utilisation of care may be a result of practice variation but also of other factors. The utilisation literature provides less (and often no) cues to explanations for practice variation. However, the MESH term “small-area analysis” (included in the PubMed search strategy) picks up some of the utilisation literature as far as it is restricted to geographic areas (usually market areas of care provision). The final search strategies are included in Appendix A.

### Screening process

**Fig. 2:**
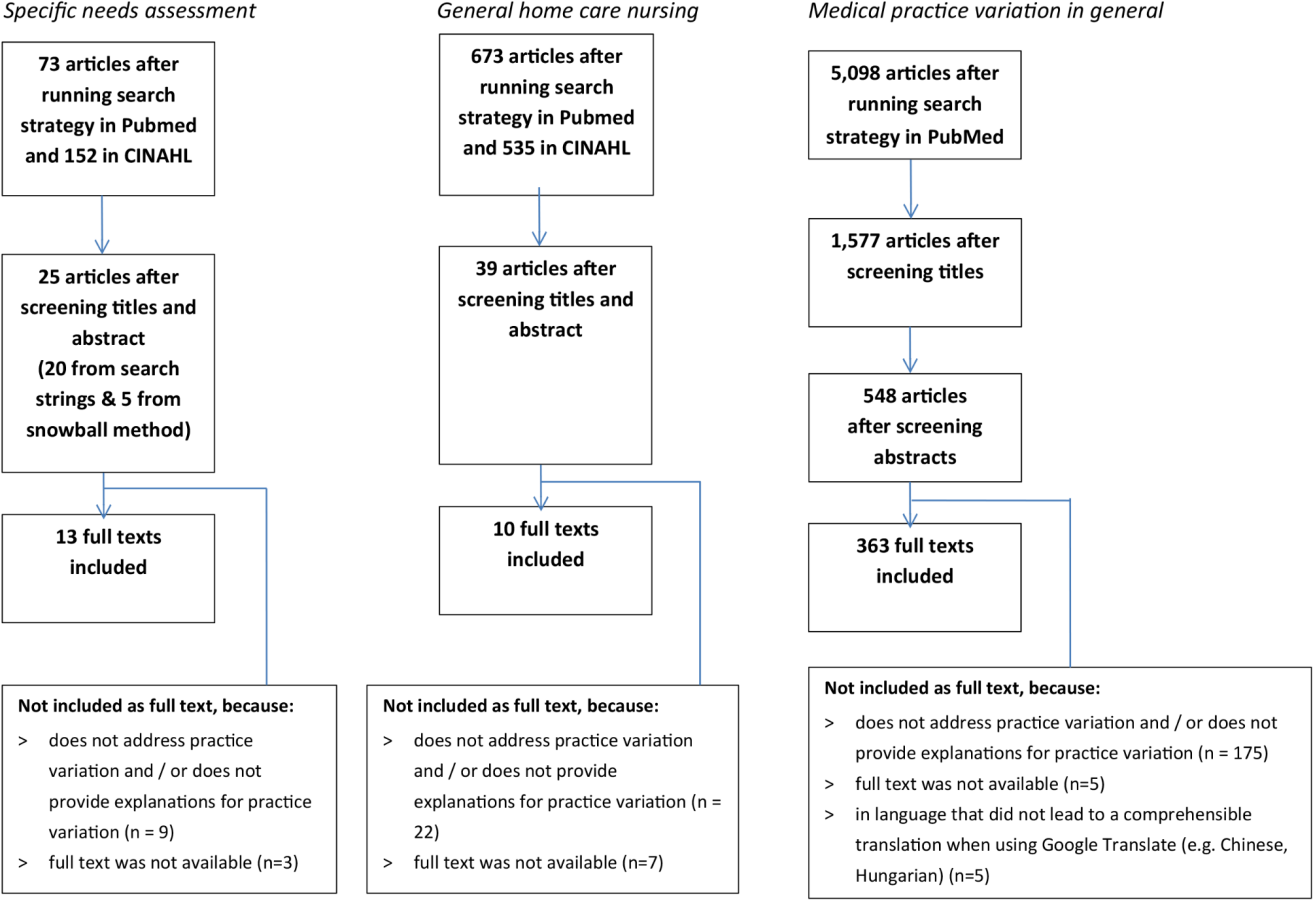
Overview of the three search strategies

Firstly, we screened the titles and abstracts of the articles found. Articles that were included in more than one search strategy were only included once. For the specific search on practice variation in needs assessment in home care nursing, the titles and abstracts were screened independently and then discussed by two researchers (AB and PG). Inclusion criteria were that the article was about variation in needs assessment in home care nursing. Due to the low number of articles, we also screened full-text articles on needs assessment that did not explicitly used key words referring to explanations for practice variation in needs assessment or mentioned it in the title or the abstract. Because of the low number of remaining articles after screening titles and abstracts, we also looked in PubMed at “similar articles” and “cited by” to find additional articles that are possibly relevant. After screening all articles, 13 were included (see also Fig. 2). Most of these were about variables related to performing needs assessments and not about explaining practice variation in needs assessment. Therefore, we continued with screening the results of the search on practice variation in home care nursing, and on practice variation in general to be able to get also insight in explanations for practice variation.

For the general search on practice variation in home care nursing, the titles were also screened independently on relevance by two researchers (PubMed AB and JDJ; CINAHL AB and PG). Subsequently, the same researchers independently screened the abstracts. The main inclusion criterion was that the article was about variation in home care nursing. For both the specific and general search, when there was no agreement between the researchers this was discussed until agreement was reached. For the search into medical practice variation in general we also first screened the titles on relevance. Because of the number of titles, three researchers (AB, PG and JDJ) independently screened 500 titles, and discussed the results of this together. Thereafter, each of the three researchers screened two thirds of the other titles. As such, two researchers screened each title. The screened titles were discussed in pairs, and when there was no agreement between the researchers this was discussed until agreement was reached. After screening the titles, the abstracts of the possibly remaining relevant articles were screened. The abstracts selected from the 500 titles that were screened first (*n* = 233) were again first screened independently by three researchers (AB, JDJ and PG) and then discussed with each other. Based on a number of criteria drawn up during the discussion of the first abstracts (see Table 1), each researcher then screened a third of the abstracts still to be screened. For all three search strategies, the full texts of the remaining possibly relevant articles were searched after screening all abstracts. When no abstract was available, we used the full text in the screening process. In this process, no appraisal of the quality of the studies was made, as is common for scoping reviews, but some studies were excluded in the search on medical practice in general on methodological grounds (see Table 1).

**Table 1:** Inclusion and exclusion criteria abstract screening search on medical practice variation in general

Inclusion criteria	• Addresses practice variation after controlling for clinically relevant differences between patients
	• Addresses potential explanations for practice variation
	• Studies based on questionnaires about what physicians would do when treating specific patients
	• Articles with broader considerations on practice variation (e.g. an article in which a model is developed, or in which an overview of literature is given, but not a systematic review)
	• Systematic reviews on practice variation
Exclusion criteria	• Protocols
	• Studies aimed at outcomes or clinical variation
	• Studies conducted in one hospital or medical centre
	• Studies that only describe practice variation, without offering possible explanations
	• Questionnaire research that is not thorough methodologically sound, e.g. an undefined population, questionnaire among conference participants, a very low response, will have not been included.
	• Studies that were in a language that was not understandable after translation by Google.

### Data extraction

The full texts were screened using a data extraction form. We tested this form in a small pilot, in which four researchers (AB, MM, JDJ and PG) screened five articles of the search on medical practice in general independently, and subsequently compared and discussed their results. This resulted in a few textual adjustments to the data extraction form to make the questions of the form more clear, after which the form was used to screen the remaining articles. To decide whether a study was to be included or not, the following two screening questions were asked in the form: (1) “Is the study about practice variation and/or can practice variation be derived from the study?” (2) “Are explanations for practice variation offered in the study?” When the answer to the first and/or second question was no, the study was excluded. On top of that, we excluded studies for which the full text was not available as well as studies that were written in a language that was not understandable after translation by Google Translate (e.g. some articles in Chinese). For the specific search on practice variation in needs assessment in home care nursing the form was slightly adjusted.

For the full texts that were included, we noted in the data extraction form the authors, title, year of study, the country where the study was carried out, the type of the study (i.e. empirical research, article with broader considerations or a synthesis of the literature), the methods used, between what units practice variation was examined (e.g. between care providers or between organisations), the mentioned explanations for the observed practice variation, and whether or not these explanations have been empirically tested. Two researchers each screened the full texts for the specific search on practice variation in needs assessment (PubMed AB and MM; CINAHL AB and JDJ), and the general search on practice variation in home care nursing (PubMed AB and JDJ; CINAHL AB and PG). Given the large amount of articles in the search on medical practice variation in general, screening of the full texts was carried out by six researchers (AB, MM, PG, JDJ, NB and SZ). When one of the researchers had doubts about the inclusion of an article, a second researcher was consulted to seek for consensus.

### Data analysis

When screening the full texts, it appeared that the majority of the articles examined empirical relations between various independent variables and practice variation instead of more general explanations for, or hypotheses about, practice variation. All these variables related to practice variation found in the literature have been included. First, two researchers (AB and MM) independently have grouped, for each search string separately, all the individual variables mentioned in the data extraction forms into categories. For example, variables like “low uptake of guidelines”, “this variation is largely due to the challenges of integrating guidelines into care”, and “conflicting guidelines” were grouped. And variables like “decreasing reimbursement rates” and “economic incentives within larger treatment centers to retain patients” were also grouped. Subsequently, AB and MM compared their results and put some subcategories for variables together into one category. For example, the variables in the category guidelines were grouped into ‘lack of guidelines’, ‘conflicting guidelines’, ‘acceptance of guidelines’ etc. Next, the categories, and subcategories, were divided into micro, meso and macro level by two researchers (AB and JDJ) in a meeting. During this meeting, AB and JDJ also classified the categories in somewhat higher levels of abstraction. For example, the categories ‘culture’, ‘norms’ and ‘opinion leaders in a region’ were grouped together in ‘customs and conventions’ (see also Tables 3 and 4).

## Results

### Characteristics of the included studies

Ultimately, 386 articles were included, namely 13 from the specific search on practice variation in needs assessment in home care nursing, 10 from the general search on practice variation in home care nursing, and 363 from the search into medical practice variation in general (see Fig. 2 for an overview of the three search strategies and Appendix B for an overview of all 386 included studies). Table 2 provides an overview of the characteristics of the included studies. From the 386 studies included, most were conducted in the United States (*n* = 162), followed by the Netherlands (*n* = 38). Approximately eight out of ten (*n* = 318) were empirical studies. The majority of the studies used medical records of patients (*n* = 91). Variation was mainly investigated between organizations (*n* = 146) and individual care providers (*n* = 145).

**Table 2:** Characteristics of the included studies of the three searches

Characteristic	Specific search on practice variation in needs assessment in home care nursing (13 articles)	General search on practice variation in home care nursing (10 articles)	Search on medical practice variation in general (363 articles)
**Year of publication**			
• 1984 - 1990	–	–	10
• 1991 - 2000	2	1	45
• 2001 - 2010	5	2	65
• 2011 – 2020	3	6	202
• 2021-2023	3	1	41
**Language in which article is written**			
• English	13	10	356
• German	–	–	3
• Spanish	–	–	2
• French	–	–	1
• Dutch	–	–	1
**Country in which study was conducted**			
• United States	3	4	155
• Canada	3	–	21
• The Netherlands	3	–	36
• Great Britain (England, UK, Northern Ireland, Scotland)	1	1	13
• Australia	–	1	9*
• New Zealand	–	–	8*
• Denmark	–	–	5
• Germany	–	–	5
• Norway	–	1	7
• Sweden	–	2	7
• Japan	–	–	6
• Other country (e.g. France, Switzerland, Italy and Spain)	2	–	21
• Multiple countries (including North America)	–	–	16
• Unknown	–	–	4
• Not applicable (e.g. review)	1	1	50
**Empirical study**			
• Yes, empirical	10	9	299
• Yes, synthesis of the literature	2	–	15
• No, article with broader considerations	1	1	49
**Method**			
• Claims data/population-wide register	–	2	37
• Hospital/medical records	2	–	89
• Survey (including vignette studies)	2	3	48
• Review	2	–	18
• Qualitative (interviews, observations)	4	1	9
• Other (e.g. a database)	1	2	67
• Combination of methods (e.g. survey and medical records, linked data)	1	1	47
• Not applicable (article with broader considerations)	1	1	48
Variation studied between**			
• Individual care providers	3	5	137
• Teams	–	–	10
• Organisations (e.g. hospitals)	1	2	143
• Regions (e.g. countries, provinces)	2	1	80
• Different (e.g. in a review)	–	–	10
• Other (e.g. two sectors)	–	2	–
• Not applicable (article with broader considerations)	–	–	32
• Not applicable (no variation measured)***	7	–	–

* One article includes data from Australia and New Zealand

** Adds to more than 363 for the search on medical practice variation in general, as a number of articles examined variation across multiple categories

*** Only applicable to specific search on practice variation in performing needs assessment in home care nursing

### Main findings

The literature searches revealed many different variables related to practice variation at different levels (see Table 3). Not surpringly, the majority of these variables were found in the search on medical practice variation in general. Some variables that relate to practice variation play a role on several levels, e.g. the availability of evidence about the effectiveness of a treatment. Lack of evidence may give room to national or regional consensus about what is good practice (macro level), to different protocols between care organisations (meso level), and the reliance on individual experience may cause variation between providers (micro level). This also applies to guidelines. Where at the macro and meso levels the availability of a guideline can play a role in variation, at the micro level not adhering to guidelines can be an explanation for variation. Variables relating to the supply-side of health care play a role as well, such as the availability of resources (in terms of both technology and personnel), the possibilities for substitution, and the number of care providers in a region or country (i.e. the local doctor density).

**Table 3:** Variables found in the literature on the macro, meso and micro level of healthcare providers*

	Specific search on practice variation in needs assessment in home care nursing (13 articles)	General search on practice variation in home care nursing (10 articles)	Search on medical practice variation in general (363 articles)
Healthcare provider macro level (regional/national)	**National and regional circumstances** • (Financial) resources (a better mix of services and resources in large-sized municipalities, differences in home-care spending between provinces, influence of insurance organisations) • Lack of regulation	**National and regional circumstances** • Supply (e.g. substitution possibilities between home care and nursing homes, number of home health agencies, rural/urban)	**National and regional circumstances** • Population characteristics (local burden of disease) • Regional strategy (local sensitivity to the problem) • Incentives (supplier induced demand theory) • Supply (local doctor density, accessibility of facilities, availability of resources (both technology and personnel), number of beds (“a bed built is a bed filled”), substitution possibilities, rural/urban) • Centralisation of complex cases in limited number of hospitals
	**Scientific evidence** • Lack of best practices • Information (e.g. decision support tools, value/benefit information, literature) • Lack of accountability guidelines		**Scientific evidence** • Evidence (lack of evidence, poor consensus, conflicting evidence, changing evidence, no learning health care system) • Diffusion of new knowledge/ technology (dissemination of skills in a region among doctors) • Guidelines (availability, conflicting guidelines (e.g. national versus regional))
	**Customs and conventions** • Culture		**Customs and conventions** • Local and national culture (surgical signature, regional attitudes, perceptions and practice patterns) • Norms (midwifery and medical relationships) • Opinion leaders in region
Healthcare provider meso level (team/organisation)	**Local circumstances** • Workload, caseload size • Organisational structure and processes	**Local circumstances** • Patient population • Champions within organisation; leadership for spread of innovation • Organizational/structural conditions (e.g. opportunities, information, support, and resources, as well as informal power and formal power). • Enviromental issues (e.g. sharing a workplace, no computer available)	**Local circumstances** • Organisation characteristics (big/ small, academic or not, teaching or not, public/private) • Size of the patient population • Policy of the organisation • Organisation of clinical pathways • Protocols and standards (content differs between organisations, degree to which written procedures are promoted, availability of and adherence to procedures, lack of feedback) • Supply (access to facilities, availability of resources (both technology and personnel), number of beds (“a bed built is a bed filled”), substitution possibilities) • Financial incentives (supplier induced demand theory, competition between hospitals) • (Inter-organisational) networks (joint decision-making among doctors, clinician networks around patients, continuity of care) • Capacity for organisational change
		**Scientific evidence** • Acceptance of evidence	**Scientific evidence** • Guidelines (acceptance, adherence to guidelines, access to guidelines, availability of resources to apply guidelines in practice (e.g. time), degree to which guideline is in line with practice) • Adopting new technologies (electronic medical records and inbuilt decision support tools, app incorporating local formularies)
	**Customs and conventions** • Culture • Interaction with peers	**Customs and conventions** • Social norms, acceptance of new roles in organisations with traditional norms	
	**Resources**,** knowledge**,** and experience** • Personnel turnover • Time that can be spent on clients versus time that needs to be spent on other priorities • Internal review of needs assessment with training • Peer review and intervention among team members	**Resources**,** knowledge**,** and experience** • Availability of personnel • Access to regular training	**Knowledge and experience** • Skills of the team (composition and experience, knowledge and skills of the staff, training) • Experience with certain patient groups
			**Habits** • Organisational culture/work context, team culture/context • Social norms (micro climate, what are colleagues doing, doing what colleagues do, social standardisation, professional interaction (with others in workplace, role, and frequency), group versus solo practice, follow the leader, influence of trainers, unclarity about roles and responsibilities) • View of role in society
Healthcare provider micro level (individual provider)	**Healthcare provider characteristics** • Gender • Age	**Healthcare provider characteristics** • Trust in technology • General acceptance of feedback (of colleagues)	**Healthcare provider characteristics** • Gender (communication style, empathy, focus on prevention by women) • Willingness to change (degree to which one is open to the opinion of others) • Psychological propensities (tolerance for uncertainty) • Professional identity
	**Work load** • Case load • Time of day, scheduling • Stresses of the many details that a home healthcare clinician experiences		**Work load** • Workload, time pressure
	**Resources**,** knowledge**,** and experience** • Experience • Skills • Education • Level of ability • Intake specialisation • Time that can be spent on clients versus time that needs to be spent on other priorities within a pressured caseload	**Knowledge and experience** • Prior experience with technology • Developing competences • Autonomy • Experiences (years, previous)	**Knowledge and experience** • Training • Competencies • Skills • Experience (in years, with a specific treatment, with specific patient groups, surgeon volume) • Educational context (who educated you, physician discipline)
	**Customs and conventions** • Intuition, perception, attitude • Interpretation (different interpretations of what is seen and of tests, different evaluation methods, nursing diagnosis in OMAHA system, understanding OASIS items may result in differences in response)	**Customs and conventions** • Practice style • Preferences	**Customs and conventions** • Preferences, beliefs, attitudes, intuition (professional autonomy, practice style, enthusiasm hypothesis, threshold hypothesis) • View of role in society
		**Scientific evidence** • Receptiveness to clinical information	**Scientific evidence** • Evidence ((lack of) acceptation of evidence, (lack of) awareness of evidence, awareness of international research, knowledge of evidence, interpretation of evidence, relying on own experience instead of evidence) • Guidelines (attitude towards guidelines, unreasoned deviation from guidelines, acceptance, adherence to guidelines, access to guidelines, availability of resources to apply guidelines in practice (e.g. time), degree to which guideline is in line with practice, treatment of a specific patient group according to guidelines ensures optimal treatment for all patients) • Adopting new technologies
		**Expectations** • Concern regarding financial issues	**Expectations** • Risk perception (risk of missing diagnosis, risk avoidance, risk awareness, legal risk) • Assess patient characteristics and expectations (discrimination based on age, gender, or ethnicity, cognitive/personal bias) • Expectations of referring providers

* Words in parentheses are the terms used in the articles

At the meso level, norms and usual practice within a team or organization play a role. In a team providers make other decisions than are made individually, indicating that there can be a ‘follow the leader’ effect or an influence of trainers. Social norms within teams and organisations can influence practice variation through peer pressure (‘doing what colleagues do’ is easier than deviating). In addition, the resources within the team or organization play a role. Resources refer to physical resources, such as available technology, as well as human resources, such as the team composition and the experience of the team, and the knowledge and skills of the individuals within the team.

On the patient side (see Table 4), the preferences of both patients (micro level) and their social context (peer/family pressure; meso level) have been suggested as explanations for practice variation, but the patients’ resources can explain part of practice variation as well (e.g. the ability to pay for care, the existence of a support network). A combination of provider and patient characteristics may influence practice variation when the clinical situation of patients and/or guidelines provide or rather restrict the opportunity to apply different treatments – the diagnosis or guideline determinedness of treatment options. The role of the partner and the social network of the patient emerged more often as a relevant variable in the literature on practice variation in needs assessment in home care nursing. Also the workload, and time as a scarce resource (i.e. time spent on one client cannot be spent on other clients and activities), emerged explicitly in this literature. Finally, two articles from the search on practice variation in needs assessment in home care nursing mentioned the steering effect of classification systems as a potential explanation for differences in needs assessment.

**Table 4:** Variables found in the literature on the macro, meso and micro level of patients*

	Specific search on practice variation in needs assessment in home care nursing (13 articles)	General search on practice variation in home care nursing (10 articles)	Search on medical practice variation in general (363 articles)
Patient macro level (health and care system; national institutions and values)			
Patient meso level (social context patient)	**Social context** • Availability, reliability and resilience of the social network/ social support system (differences in the caregivers characteristics, family support, current level of informal and formal care, access to informal care, number of children) • Preferences/influence of informal caregivers • Team of healthcare providers around the patient	**Social context** • Social context of the patient	**Social context** • Family/peer pressure • Family/peer support • Regional index of need
Patient micro level (individual patient)	**Patient characteristics** • Clinically relevant characteristics (health status, care/client needs, differences in characteristics of the older persons, marital status, SES, coping, risk to client and family, age and gender (higher age and women means more care), the presence of ADL limitations and externalizing behaviours, cognitive disability, nutritional status, living situation, recent termination of services, activities of daily living • Cooperation of the client, client cues		**Patient characteristics** • Clinical relevant characteristics • Social and cultural background (socio-economic status, related to coping style) • Type of insurance of the patient
	**Preferences** • Preferences • Beliefs		**Preferences** • Preferences and pressure • Expectations • Beliefs (cultural, historical, religious)
	**Personal resources** Clients’ capacity for self-reliance and self-direction		**Resources** • Knowledge • Skills • Health literacy • Income (ability to pay for care)

* Words in parentheses are the terms used in the articles

## Discussion

Our study aimed to get insight into potential explanations for practice variation in needs assessment by home care nurses from the published literature. Because of a lack of articles that discuss explanations in this specific area, we went to the broader literature on practice variation in order to transfer these explanations to practice variation in needs assessments by home care nurses. To get insight into potential explanations, a scoping review of the international literature was performed.

We found a wide variety of variables that are empirically related to practice variation and that might have a role in explaining practice variation at different levels, from individual care providers to the teams and organisations they work in and the health system as a whole. Variables relating to patients and their social context are more present in the literature on needs assessments performed by home care nurses than in the literature on care practice by home care nurses or on practice variation in general. The context of patients concerns, for example, their living situation, the role of their partner, and the social network of patients.

### Interpretation of the main findings

#### Little research on variation in nursing practice

As expected, we found much less published literature on practice variation in nursing than on medical practice variation. There is a much longer tradition of research in this area in medical care than in nursing care. Medical practice variation was already frequently studied since the 1970’s. Still, much of the research we reviewed was performed in the USA. As we were looking for general explanations for practice variation and to the potential transfer to the situation of needs assessment in home care nursing (and ultimately in testing these potential explanations in new research), we don’t see this as an important limitation. Moreover, the organisation and provision of home care also differs strongly between European countries. Nurses have long been seen and treated as supporting staff for doctors [18] and still often are, i.e. nurses helping doctors instead of nurses helping patients. If nurses are not seen as autonomous professionals who make their own professional choices in the support and treatment of patients, it is understandable that the idea and the relevance of studying variation in their patient care and treatment choices does not come up easily.

#### From variables to mechanisms

In the literature, we found mainly variables that play a role in explaining practice variation in general. We tried to group these variables in broader categories, but important are the mechanisms behind these groups of variables that explain practice variation. Part of the variables that are related to practice variation relate to clinical differences between patients. These are of interest in determining whether or not practice variation is warranted. Other variables are related to organisational and social mechanisms that operate at the different levels we have distinguished. We derived these mechanisms from general insights from the social sciences (see for example De Jong [7]). At the *macro* level of national and regional care systems, the following plays a role (see also Table 5 for an overview), (1) institutions (the written or unwritten rules of the care systems); (2) structures, that is about the relationships between parts of the system (e.g. home care and hospital care), and the position of different groups in society (e.g. the position and autonomy of nurses vis-à-vis doctors); (3) resources (e.g. the availability of personnel, technology and budget) and (4) lack of evidence about effectiveness of care. At *meso* level of organisations and teams within organisations, the above mentioned macro level mechanisms have their impact on how things are usually done, on the positions of different groups in the organisations, and the positions within teams and on their resources. This is extended with specific mechanisms at the meso level that relate to organisational and group processes (e.g. the development of ideas of what is good practice, availability and distribution of information, group pressures, knowledge and experience of the team, and the policy of the organisation). At *micro* level of individual care providers and patients the macro and meso level mechanisms have their impact and these are extended with specific mechanisms at this level that relate to the action formation of providers and patients (e.g. their individual preferences, expectations and endowments, such as education, income etc.) and the interaction situation (e.g. the degree to which patients are able to express their preferences).

**Table 5:** An overview of mechanisms explaining practice variation at micro, meso and macro level

Level	Explanatory mechanisms	Examples of variables from our review	Examples of applying mechanisms to needs assessment in home care
Macro: National or regional care system	Institutions Structures Resources Lack of evidence	‘Regional strategy’ ‘Local doctor density’ ‘Availability of guidelines’ ‘Resources (both technology and personnel)’	- The steering influence of standardized nursing terminology, an example of a cultural-cognitive mechanism - Framework to adhere to when performing needs assessments (in Dutch: normenkader), as an example of a normative mechanism
Meso: Organisation and team and social context of the patient	Impact of the previous level + organisational and group process	‘Doing what colleagues do’ ‘Skills of the team’ ‘Organisational culture’ ‘Social context of patients’	- Adaptation towards the norms that apply in the team and organisation - The patient and the social context of the patient; as a needs assessment looks what patients can do themselves and what their context can contribute
Micro: Healthcare provider and patient	Impact of the previous levels + individual preferences and endowments, interaction situation	‘Adherence to guidelines’ ‘Experience (e.g. in years)’ ‘Skills’ (of both provider and patient) ‘Practice style’ ‘Beliefs (cultural, historical, religious)’ ‘Preferences of patients’	- The patient and the social context of the patient; as a needs assessment looks what patients can do themselves and what their context can contribute

#### Application of potential explanations to the situation of home care nursing

The specific contribution of this paper is the attention to practice variation in the home nursing profession and in the transfer of the findings, described above from the broad literature on medical practice variation, to the situation of home care nursing. The results of our review cannot be applied to home care nursing directly, as there are differences between medical care and home care. For example, the main goal of medical care is the treatment or recovery of a patient with traditionally, mostly, an acute condition/disease, whereas home care is focused on care needed to allow patients to live at home as well as possible [18, 19]. Moreover, medical care is mainly provided in practices and hospitals, while home care nursing is provided at the patient’s home. The consequence of this is that the social context (e.g. the living situation in a broad sense) of patients is more prominent. Furthermore, doctors make by tradition their decisions autonomously (i.e. having individual authority and freedom to make decisions concerning the patient [20]). In the Netherlands, the role of home care nurses changed over the years. In the past, they formed a broad and autonomous profession with respect to prevention, nursing, and care of the vulnerable. With the emergence of larger home care organisations and job differentiation in 1980s and 1990s, this changed. Since 2015, home care nurses have again more professional autonomy, among others, due to the rise of self-organising teams [19].

Taking into this account, we will provide examples of how mechanisms at the micro, meso and macro level play a role in explaining variation in needs assessment (see also Table 5). At the *micro* level, home care nurses perform needs assessment for individual patients. This assessment is aimed at strengthening the patients’ own direction and self-reliance and the patients’ social support system: it looks at what patients can do themselves and what their social context can contribute. As such, patients, their preferences and their social context are expected to play a more important role in explaining variation in home care than in medical care. At the *meso* level, adaptation is expected to play a role in home care nurses. Most home care nurses work in teams within organisations, and it can be reasoned that nurses, when performing needs assessments, adapt to the norms that apply in their team and organisation. At the *macro* level, guidelines, as an example of a normative mechanism, are expected to play a role. They contain recommendations that help care providers in their clinical decision-making and are based on scientific research, supplemented with expertise and experiences from care providers and patients. It is suggested that guidelines reduce variation when more care providers follow them [21, 22]. In the Netherlands, guidelines and standards for nurses exist, but they are most often not aimed at home care nursing. And if a guideline mentions home care nursing explicitly, little attention is paid to the practice of home care nurses [23]. This may be caused by the fact that for much of nursing care no evidence is available. As a result, much care is provided on the basis of consensus. This leads to a situation where standardisation of care processes is not built on evidence-based guidelines. As a consequence, the focus will probably be more on accountability standardisation. The influence of standardized nursing terminology, an example of a cultural-cognitive mechanism, can play a role in this. Such terminology helps home care nurses to better choose, sort, and record the actions and outcomes of the care for patients. Our scoping review revealed the different interpretation by nurses of these systems of nursing terminology as a potential explanation of differences in needs assessment.

### Strengths and limitations

A strength of this scoping review is that we developed three different search strategies, which were focused on practice variation in needs assessment, on practice variation in home care nursing, and on practice variation in general. The three strategies resulted in over 6,000 references that were all assessed. Therefore, likely few references were missed. However, we only have searched for scientific articles and disregarded grey literature. This may be a limitation with regard to the specific search strategy on practice variation in needs assessment. For this search strategy less unambiguous search terms were available to search in the international literature. For example, because the person, in terms of education and function, who performs the needs assessment differs between countries.

By concentrating on practice variation in (or applicable to) home care nursing we have left out two broader areas of literature. First of all that on social care and welfare services. We also left out the literature on long-term care which may include home care nursing, but often focuses on resource allocation. Resource allocation and prioritisation may explain some of the differences between municipalities; however, the role of individual (or teams of) professionals is not highlighted. Therefore, we decided not to include that literature.

We have not addressed the question whether existence of practice variation is good or bad. Insight in the underlying causes of variation is needed to determine whether variation is warranted or not. As little is known about practice variation among home care nurses, it is important to examine the causes of practice variation in needs assessment before specific interventions and policy aimed at reducing practice variation can be developed [24].

Despite these limitations, we believe this scoping review contributes to the knowledge of an area of research and practice that has been rarely studied before.

## Conclusions

Our scoping review found almost no research into describing and explaining practice variation in home care nursing. This scoping review, however, gave insight into a wide variety of variables at the micro, meso, and macro level that might play a role in explaining practice variation in general. We argued how these variables are related to mechanisms, and gave some examples of how these mechanisms can be applied to home care nursing, and more specifically to practice variation in needs assessment. A next, future step is to empirically examine the role that these mechanisms play in explaining practice variation in needs assessment.

### Electronic supplementary material

Below is the link to the electronic supplementary material.

Supplementary Material 1

## Data Availability

Not applicable.
