# Supplementary material for: Practice variation in home care nursing: mapping potential explanations through a scoping review of the literature
Source: Res Health Serv Reg. 2024 Aug 21;3:12. doi: 10.1007/s43999-024-00048-8 (PMC11336153; doi:10.1007/s43999-024-00048-8)
Supplement: Supplementary file 1 — Supplementary Material 1 [file 43999_2024_48_MOESM1_ESM.docx]

**APPENDIX A – SEARCH STRATEGIES IN PUBMED AND CINAHL**

**Search strategy practice variation in needs assessment in home care nursing Pubmed (run on June 5, 2023; 73 articles)**

(home care nurs*[TIAB] OR home health nurs*[TIAB] OR home nurs*[TIAB] OR district nurs*[TIAB] OR community nurs*[TIAB] or community health nurs*[TIAB] or home visiting nurs*[TIAB] OR home care[TIAB] OR community care[TIAB] OR long term care[TIAB] OR primary nursing care[TIAB] OR domiciliary care[TIAB] OR Home Care Services[MeSH] OR Home Care Service*[tiab] OR community care service*[TIAB] OR home health care[TIAB] OR home healthcare[TIAB] or home based[TIAB] OR home setting*[TIAB] OR domestic care[TIAB] OR domestic healthcare[TIAB] OR domestic health care[Title/Abstract]) **AND** (need assessment*[TIAB] OR needs assessment*[TIAB] OR needs assessment[MeSH] OR care assessment*[TIAB] OR care needs assessment*[TIAB] OR formal assessment*[TIAB] OR Determination of Health Care Need*[Title/Abstract] OR Determination of Healthcare Need*[Title/Abstract] OR Assessment of Health Care Needs[Title/Abstract] OR Assessment of HealthCare Needs[Title/Abstract]) **AND** (variation*[TIAB] OR Small-Area Analysis[MeSH] OR small area analys*[tiab] OR Practice Patterns, Nurses'[MesH] OR nurse practice pattern*[tiab] OR nurses practice pattern*[tiab] OR Practice Patterns, Physicians’[MeSH] OR physician practice pattern*[tiab] OR physicians practice pattern*[tiab])

**Search strategy practice variation in needs assessment in home care nursing CINAHL (run on June 19, 2023 (151 articles)**

(MH (Home Health Care) OR TI (home care nurs* OR home health nurs* OR home nurs* OR district nurs* OR community nurs* OR community health nurs* OR home visiting nurs* OR home care OR community care OR long term care OR primary nursing care OR domiciliary care OR home care service OR community care service OR home health care OR home healthcare OR home based OR home setting OR domestic care OR domestic healthcare OR domestic health care) OR AB (home care nurs* OR home health nurs* OR home nurs* OR district nurs* OR community nurs* OR community health nurs* OR home visiting nurs* OR home care OR community care OR long term care OR primary nursing care OR domiciliary care OR home care service OR community care service OR home health care OR home healthcare OR home based OR home setting OR domestic care OR domestic healthcare OR domestic health care)) **AND**

(MH (needs assessment) OR TI (need assessment* OR needs assessment* OR care assessment* OR care needs assessment* OR formal assessment* OR Determination of Health Care Need* OR Assessment of Health Care Needs OR Assessment of HealthCare Needs) OR AB (need assessment* OR needs assessment* OR care assessment* OR care needs assessment* OR formal assessment* OR Determination of Health Care Need* OR Assessment of Health Care Needs OR Assessment of HealthCare Needs)) **AND**

MH (Practice patterns) OR TI (variation* OR small area analys* OR nurse practice pattern* OR nurses practice pattern* OR physician practice pattern* OR physicians practice pattern*) OR AB (variation* OR small area analys* OR nurse practice pattern* OR nurses practice pattern* OR physician practice pattern* OR physicians practice pattern*))

**Search strategy practice variation in home care nursing Pubmed (run on June 19, 2023; 673 articles)**

(home care nurs*[TIAB] OR home health nurs*[TIAB] OR home nurs*[TIAB] OR district nurs*[TIAB] OR community nurs*[TIAB] or community health nurs*[TIAB] or home visiting nurs*[TIAB] OR home care[TIAB] OR community care[TIAB] OR long term care[TIAB] OR primary nursing care[TIAB] OR domiciliary care[TIAB] OR Home Care Services[MeSH] OR Home Care Service*[tiab] OR community care service*[TIAB] OR home health care[TIAB] OR home healthcare[TIAB] or home based[TIAB] OR home setting*[TIAB] OR domestic care[TIAB] OR domestic healthcare[TIAB] OR domestic health care[Title/Abstract]) **AND** (practice variation*[TIAB] OR Small-Area Analysis[MeSH] OR small area analys*[tiab] OR Practice Patterns, Nurses'[MesH] OR nurse practice pattern*[tiab] OR nurses practice pattern*[tiab] OR Practice Patterns, Physicians’[MeSH] OR physician practice pattern*[tiab] OR physicians practice pattern*[tiab])

**Search strategy practice variation in home care nursing CINAHL (run on June 19, 2023; 535 articles)**

MH (Home Health Care) OR TI (home care nurs* OR home health nurs* OR home nurs* OR district nurs* OR community nurs* OR community health nurs* OR home visiting nurs* OR home care OR community care OR long term care OR primary nursing care OR domiciliary care OR home care service OR community care service OR home health care OR home healthcare OR home based OR home setting OR domestic care OR domestic healthcare OR domestic health care) OR AB (home care nurs* OR home health nurs* OR home nurs* OR district nurs* OR community nurs* OR community health nurs* OR home visiting nurs* OR home care OR community care OR long term care OR primary nursing care OR domiciliary care OR home care service OR community care service OR home health care OR home healthcare OR home based OR home setting OR domestic care OR domestic healthcare OR domestic health care)

**AND**

MH (Practice patterns) OR TI (practice variation* OR small area analys* OR nurse practice pattern* OR nurses practice pattern* OR physician practice pattern* OR physicians practice pattern*) OR AB (practice variation* OR small area analys* OR nurse practice pattern* OR nurses practice pattern* OR physician practice pattern* OR physicians practice pattern*)

**Search strategy on medical practice variation in general Pubmed (run on June 19, 2023; 5,098 articles)**

Practice variation*[TIAB] OR Clinical variation*[TIAB] OR Small-Area Analysis[MeSH] OR Practice Pattern[TIAB]

**APPENDIX B – OVERVIEW OF THE INCLUDED STUDIES OF THE SEARCH STRATEGIES**

*Specific search on practice variation in needs assessment in home care nursing*

1. Moshabela M, Sips I, Barten F. Needs assessment for home-based care and the strengthening of social support networks: the role of community care workers in rural South Africa. Glob Health Action. 2015 Dec 18;8:29265.
2. Peckham A, Williams AP, Neysmith S. Balancing formal and informal care for older persons: how case managers respond. Can J Aging. 2014 Jun;33(2):123-36.
3. Fraser KD, Estabrooks C, Allen M, Strang V. Factors that influence case managers' resource allocation decisions in pediatric home care: an ethnographic study. International journal of Nursing Studies. 2009;46:337-349.
4. Fraser KD, Estabrooks C. What factors influence case managers' resource allocation decisions? A systematic review of the literature. Decision making in clinical practice. 2008 May;28(3):394-410.
5. Patnaik A, Elliott TR, Moudouni DM, Fournier CJ, Naiser E, Miller TR, Dyer JA, Hawes C, Phillips CD. Severity of children's intellectual disabilities and Medicaid personal care services. Rehabil Psychol. 2011 Nov;56(4):383-90.
6. Shew PA, Sanders SL, Arthur NC, Bush KW. OASIS inter-rater reliability and reimbursement: a study of inter-rater reliability of the Outcome and Assessment Information Set (OASIS): its effects on the Home Health Resource Group (HHRG) and reimbursement. Home Healthc Nurse. 2010 Jan;28(1):31-6.
7. Hammar T, Perälä ML, Rissanen P. Clients' and workers' perceptions on clients' functional ability and need for help: home care in municipalities. Scand J Caring Sci. 2009 Mar;23(1):21-32.
8. Coyte PC, McKeever P. Home care in Canada: passing the buck. Can J Nurs Res. 2001 Sep;33(2):11-25.
9. Cowley S, Bergen A, Young K, Kavanagh A. A taxonomy of needs assessment, elicited from a multiple case study of community nursing education and practice. J Adv Nurs. 2000 Jan;31(1):126-34.
10. Marek KD. Nursing diagnoses and home care nursing utilization. Public Health Nurs. 1996 Jun;13(3):195-200.
11. Brabers AEM, De Groot K, Groenewegen PP, De Jong JD. To what extent do home care nurses feel free to assess the care that is needed for their patients? A nationwide survey in the Netherlands. Health Sci Rep. 2021;4:e420.
12. Schwenke M, Van Dorst J, Zwakhalen S, De Jong JD, Brabers AEM, Bleijenberg N. Measures to improve patient needs assessments and reduce practice variation in Dutch home care organizations. Nursing Open. 2023;10:3052–3063.
13. Van Dorst JIE, Schwenke M, Bleijenberg N, De Jong JD, Brabers AEM, Zwakhalen SMG. Defining practice variation and exploring influencing factors on needs assessment in home care nursing: A Delphi study. Journal of Advanced Nursing 2023. DOI: 10.1111/jan.15680

*General search on practice variation in home care nursing*

1. Brabers AEM, de Groot K, Groenewegen PP. Practice variation among home care nurses. Prim Health Care Res Dev. 2019 Oct 1;20:e136.
2. Huang SS, Kim H. Home Health Chains and Practice Patterns: Evidence of 2008 Medicare Reimbursement Revision. Population Health Management. 2017 https://doi.org/10.1089/pop.2016.0107
3. Thompson C, Adderley U. Diagnostic and treatment decision making in community nurses faced with a patient with possible venous leg ulceration: a signal detection analysis. Int J Nurs Stud. 2015 Jan;52(1):325-33. doi: 10.1016/j.ijnurstu.2014.10.015.
4. Torvik K, Nordtug B, Brenne IK, Rognstad MK. Pain Assessment Strategies in Home Care and Nursing Homes in Mid-Norway: A Cross-sectional Survey. Pain Manag Nurs. 2015 Aug;16(4):602-8. doi: 10.1016/j.pmn.2015.01.001
5. Blanck S, Engström M. District nurses’ prescribing practice and its link to structural conditions. J Am Assoc Nurse Pract. 2015 Oct;27(10):568-75. doi: 10.1002/2327-6924.12234.
6. Courtney-Pratt H, Cummings E, Turner P, Cameron-Tucker H, Wood-Baker R, Walters EH, Robinson AL. Entering a world of uncertainty: community nurses' engagement with information and communication technology Comput Inform Nurs. 2012 Nov;30(11):612-9. doi: 10.1097/NXN.0b013e318266caab.
7. Fortinsky RH, Baker D, Gottschalk M, King M, Trella P, Tinetti ME. Extent of implementation of evidence-based fall prevention practices for older patients in home health care. J Am Geriatr Soc. 2008 Apr;56(4):737-43. doi: 10.1111/j.1532-5415.2007.01630.x.
8. Brega AG, Jordan AK, Schlenker RE. Practice variations in home health care. Home Health Care Serv Q. 2003;22(3):41-64.
9. Kenney GM, Dubay LC. Explaining area variation in the use of Medicare home health services. Med Care. 1992 Jan;30(1):43-57.
10. Löyttynen J, Graneheim UH, Hörnsten Å. District Nurses’ Experiences of Practice in Caring for People with Mental Ill-Health in Swedish Primary Care. Issues in Mental Health Nursing, 2023: 44:5, 396-405.

*Search on medical practice variation in general*

1. Okubo Y, Miura M, Kobayashi T, Morisaki N, Michihata N, Matsui H, Fushimi K, Yasunaga H. The Impact of Changes in Clinical Guideline on Practice Patterns and Healthcare Utilizations for Kawasaki Disease in Japan. Front Pediatr. 2020 Mar 24;8:114.
2. de Man Y, Groenewoud S, Oosterveld-Vlug MG, Brom L, Onwuteaka-Philipsen BD, Westert GP, Atsma F. Regional variation in hospital care at the end-of-life of Dutch patients with lung cancer exists and is not correlated with primary and long-term care. Int J Qual Health Care. 2020 Mar 18.
3. Hutcheon JA, Chapinal N, Skoll A, Au N, Lee L. Inter-hospital variation in use of obstetrical blood transfusion: a population-based cohort study. BJOG. 2020 Mar 9.
4. Huijben JA, Wiegers EJA, Lingsma HF, Citerio G, Maas AIR, Menon DK, Ercole A, Nelson D, van der Jagt M, Steyerberg EW, Helbok R, Lecky F, Peul W, Birg T, Zoerle T, Carbonara M, Stocchetti N; CENTER-TBI investigators and participants. Changing care pathways and between-center practice variations in intensive care for traumatic brain injury across Europe: a CENTER-TBI analysis. Intensive Care Med. 2020 Feb 25.
5. Tourlamain G, Garcia-Puig R, Gutiérrez-Junquera C, Papadopoulou A, Roma E, Kalach N, Oudshoorn J, Sokollik C, Karolewska-Bochenek K, Oliva S, Strisciuglio C, Bauraind O, Auth MK, Thomson M, Otte S, Rok O, Dias JA, Tzivinikos C, Urbonas V, Kostovski A, Zevit N, Velde SV; ESPGHAN EGID Working group. Differences in Management of Eosinophilic Esophagitis in Europe: an Assessment of Current Practice. J Pediatr Gastroenterol Nutr. 2020 Feb 24.
6. Vu JV, Sheetz KH, De Roo AC, Hiatt T, Hendren S. Variation in colectomy rates for benign polyp and colorectal cancer. Surg Endosc. 2020 Feb 19.
7. Keikes L, Koopman M, Stuiver MM, Lemmens VEPP, van Oijen MGH, Punt CJA. Practice variation on hospital level in the systemic treatment of metastatic colorectal cancer in The Netherlands: a population-based study. Acta Oncol. 2020 Apr;59(4):395-403.
8. Soni P, Rosenbaum BP, Kelly ML. Regional and Institutional Practice Variations in Decompressive Spine Surgery for Patients with Penetrating Spinal Injury in the United States. World Neurosurg. 2020 Jan 28.
9. Rodin D, Chien AT, Ellimoottil C, Nguyen PL, Kakani P, Mossanen M, Rosenthal M, Landrum MB, Sinaiko AD. Physician and facility drivers of spending variation in locoregional prostate cancer. Cancer. 2020 Apr 15;126(8):1622-1631.
10. Chang CY, Obermeyer Z. Association of Clinical Characteristics With Variation in Emergency Physician Preferences for Patients. JAMA Netw Open. 2020 Jan 3;3(1):e1919607.
11. Harrison R, Hinchcliff RA, Manias E, Mears S, Heslop D, Walton V, Kwedza R. Can feedback approaches reduce unwarranted clinical variation? A systematic rapid evidence synthesis. BMC Health Serv Res. 2020 Jan 16;20(1):40.
12. Johnson LM, White SK, Greene DN, Schmidt RL. Bad Tests Die Slowly: The Myelin Basic Protein Example. J Appl Lab Med. 2019 Dec 6.
13. Woods-Hill CZ, Koontz DW, King AF, Voskertchian A, Colantuoni EA, Miller MR, Fackler JC, Bonafide CP, Milstone AM, Xie A; Bright Star Authorship group. Practices, Perceptions, and Attitudes in the Evaluation of Critically Ill Children for Bacteremia: A National Survey. Pediatr Crit Care Med. 2020 Jan;21(1):e23-e29.
14. von Meyenfeldt EM, Hoeijmakers F, Marres GMH, van Thiel ERE, Marra E, Marang-van de Mheen PJ, Schreurs HWH. Variation in length of stay after minimally invasive lung resection: a eflection of perioperative care routines? Eur J Cardiothorac Surg. 2020 Apr 1;57(4):747-753.
15. O'Byrne ML, Millenson ME, Grady CB, Huang J, Bamat NA, Munson DA, Song L, Dori Y, Gillespie MJ, Rome JJ, Glatz AC. Trends in transcatheter and operative closure of patent ductus arteriosus in neonatal intensive care units: Analysis of data from the Pediatric Health Information Systems Database. Am Heart J. 2019 Nov;217:121-130.
16. Chen FW, LeBrett WG, Yang L, Chang L. Opioid Prescription Patterns Among US Gastroenterologists From 2013 to 2017. Gastroenterology. 2020 Feb;158(3):776-779.
17. Schang L, Koller D, Franke S, Sundmacher L. Exploring the role of hospitals and office-based physicians in timely provision of statins following acute myocardial infarction: a secondary analysis of a nationwide cohort using cross-classified multilevel models. BMJ Open. 2019 Oct 16;9(10):e030272.
18. Gilligan TC, Cook AD, Hosmer DW, Hunter DC, Vernon TM, Weinberg JA, Ward J, Rogers FB. Practice Variation in Vena Cava Filter Use Among Trauma Centers in the National Trauma Database. J Surg Res. 2020 Feb;246:145-152.
19. Wu SY, Terrell J, Park A, Perrier N. Understanding Thyroidectomy Cost Variations Among National Cancer Institute-Designated Cancer Centers. World J Surg. 2020 Feb;44(2):385-392.
20. Richards JM, Burgon TB, Tamondong-Lachica D, Bitran JD, Liangco WL, Paculdo DR, Peabody JW. Reducing Unwarranted Oncology Care Variation Across a Clinically Integrated Network: A Collaborative Physician Engagement Strategy. J Oncol Pract. 2019 Dec;15(12):e1076-e1084.
21. Pollmanns J, Drösler SE, Geraedts M, Weyermann M. Predictors of hospitalizations for diabetes in Germany: an ecological study on a small-area scale. Public Health. 2019 Dec;177:112-119.
22. Gravesteijn BY, Sewalt CA, Ercole A, Lecky F, Menon D, Steyerberg EW, Maas AIR, Lingsma HF, Klimek M; CENTER-TBI collaborators. Variation in the practice of tracheal intubation in Europe after traumatic brain injury: a prospective cohort study. Anaesthesia. 2020 Jan;75(1):45-53.
23. Jasuja GK, Engle RL, Skolnik A, Rose AJ, Male A, Reisman JI, Bokhour BG. Understanding the Context of High- and Low-Testosterone Prescribing Facilities in the Veterans Health Administration (VHA): a Qualitative Study. J Gen Intern Med. 2019 Nov;34(11):2467-2474.
24. Mann M, Wang P, Schul M, Khilnani NM, Park A, Makary MA, Hicks CW. Significant physician practice variability in the utilization of endovenous thermal ablation in the 2017 Medicare population. J Vasc Surg Venous Lymphat Disord. 2019 Nov;7(6):808-816.
25. O'Rourke T, Kirk J, Duff E, Golonka R. A survey of nurse practitioner controlled drugs and substances prescribing in three Canadian provinces. J Clin Nurs. 2019 Dec;28(23-24):4342-4356.
26. Morikawa M, Hagiwara Y, Gibo K, Goto T, Watase H, Hasegawa K; JEAN‐3 Investigators. Methylxanthine use for acute asthma in the emergency department in Japan: a multicenter observational study. Acute Med Surg. 2019 Apr 1;6(3):279-286.
27. Desveaux L, Saragosa M, Kithulegoda N, Ivers NM. Family Physician Perceptions of Their Role in Managing the Opioid Crisis. Ann Fam Med. 2019 Jul;17(4):345-351.
28. Yurso M, Box B, Burgon T, Hauck L, Tagg K, Clem K, Paculdo D, Acelajado MC, Tamondong-Lachica D, Peabody JW. Reducing Unneeded Clinical Variation in Sepsis and Heart Failure Care to Improve Outcomes and Reduce Cost: A Collaborative Engagement with Hospitalists in a MultiState System. J Hosp Med. 2019 Jun 19;14:E1-E6.
29. Bucholc M, O'Kane M, Mullan C, Ashe S, Maguire L. Primary care use of laboratory tests in Northern Ireland's Western Health and Social Care Trust: a cross-sectional study. BMJ Open. 2019 Jun 21;9(6):e026647.
30. Hicks CW, Holscher CM, Wang P, Black JH 3rd, Abularrage CJ, Makary MA. Overuse of early peripheral vascular interventions for claudication. J Vasc Surg. 2020 Jan;71(1):121-130.
31. Nanna MG, Navar AM, Wang TY, Li S, Virani SS, Li Z, Robinson JG, Roger VL, Wilson PWF, Goldberg AC, Koren A, Louie MJ, Peterson ED. Practice-level variation in statin use and low-density lipoprotein cholesterol control in the United States: Results from the Patient and Provider Assessment of Lipid Management (PALM) registry. Am Heart J. 2019 Aug;214:113-124.
32. Braun AL, Prati E, Martin Y, Dvořák C, Tal K, Biller-Andorno N, Bulliard JL, Cornuz J, Selby K, Auer R. Variation in colorectal cancer testing between primary care physicians: a cross-sectional study in Switzerland. Int J Public Health. 2019 Sep;64(7):1075-1083.
33. Johnson D, Ouenes O, Letson D, de Belen E, Kubal T, Czarnecki C, Weems L, Box B, Paculdo D, Peabody J. A Direct Comparison of the Clinical Practice Patterns of Advanced Practice Providers and Doctors. Am J Med. 2019 Nov;132(11):e778-e785.
34. Sutherland K, Levesque JF. Unwarranted clinical variation in health care: Definitions and proposal of an analytic framework. J Eval Clin Pract. 2019 May 28.
35. Piper KN, Baxter KJ, Wetzel M, McCracken C, Travers C, Slater B, Cairo SB, Rothstein DH, Cina R, Dassinger M, Bonasso P, Lipskar A, Denning NL, Huang E, Shah SR, Cunningham ME, Gonzalez R, Kauffman JD, Heiss KF, Raval MV; Pediatric Surgical Research Collaborative - PedSRC. Provider education decreases opioid prescribing after pediatric umbilical hernia repair. J Pediatr Surg. 2019 May 11.
36. Jung KYK, Shadbolt B, Rezo A. Temporal impact of the publication of guidelines and randomised evidence on the adoption of hypofractionated whole breast radiotherapy for early-stage breast cancer. J Med Imaging Radiat Oncol. 2019 Aug;63(4):530-537.
37. Wang P, Hutfless SM, Shin EJ, Hartman C, Disney S, Fain CC, Bull-Henry KP, Daniels DK, Abdi T, Singh VK, Kalloo AN, Makary MA. Same-Day vs Different-Day Elective Upper and Lower Endoscopic Procedures by Setting. JAMA Intern Med. 2019 Jul 1;179(7):953-963.
38. Albertini JG, Wang P, Fahim C, Hutfless S, Stasko T, Vidimos AT, Leshin B, Billingsley EM, Coldiron BM, Bennett RG, Marks VJ, Park A, Overton HN, Bruhn WE, Xu T, Krishnan A, Makary MA. Evaluation of a Peer-to-Peer Data Transparency Intervention for Mohs Micrographic Surgery Overuse. JAMA Dermatol. 2019 May 5.
39. Oravetz P, White CJ, Carmouche D, Swan N, Donaldson J, Ruhl R, Valdenor C, Paculdo D, Tran M, Peabody J. Standardising practice in cardiology: reducing clinical variation and cost at Ochsner Health System. Open Heart. 2019 Mar 22;6(1).
40. Okubo Y, Hayakawa I, Nariai H, Michihata N, Matsui H, Fushimi K, Yasunaga H. Recent practice patterns in diagnostic procedures anticonvulsants, and antibiotics for children hospitalized with febrile seizure. Seizure. 2019 Apr;67:52-56.
41. Perino AC, Fan J, Askari M, Heidenreich PA, Keung E, Raitt MH, Piccini JP, Ziegler PD, Turakhia MP. Practice Variation in Anticoagulation Prescription and Outcomes After Device-Detected Atrial Fibrillation. Circulation. 2019 May 28;139(22):2502-2512.
42. Dizon MLV, Rao R, Hamrick SE, Zaniletti I, DiGeronimo R, Natarajan G, Kaiser JR, Flibotte J, Lee KS, Smith D, Yanowitz T, Mathur AM, Massaro AN. Practice variation in anti-epileptic drug use for neonatal hypoxic-ischemic encephalopathy among regional NICUs. BMC Pediatr. 2019 Feb 27;19(1):67.
43. Harris AHS, Meerwijk EL, Kamal RN, Sears ED, Hawn M, Eisenberg D, Finlay AK, Hagedorn H, Marshall N, Mudumbai SC. Variation in Surgeons' Requests for General Anesthesia When Scheduling Carpal Tunnel Release. Hand (N Y). 2019 Feb 21:1558944719828006.
44. Hampson LA, Odisho AY, Meng MV. Variation in Laparoscopic Nephrectomy Surgical Costs: Opportunities for High Value Care Delivery. Urol Pract. 2018 Sep;5(5):334-341.
45. McDowell RD, Bennett K, Moriarty F, Clarke S, Barry M, Fahey T. Prescriber Variation in Relation to Prescribing Trends within the Preferred Drugs Initiative in Ireland (2012-2015): An Interrupted Time-Series Study Using Latent Curve Models. Med Decis Making. 2019 Apr;39(3):278-293.
46. Cook DA, Pankratz VS, Pencille LJ, Dupras DM, Linderbaum JA, Wilkinson JM. Associations Among Practice Variation, Clinician Characteristics, and Care Algorithm Usage: A Multispecialty Vignette Study. Am J Med Qual. 2019 Nov/Dec;34(6):596-606.
47. Vromen T, Peek N, Abu-Hanna A, Kemps HM. Practice Variations in Exercise Training Programs in Dutch Cardiac Rehabilitation Centers: Prospective, Observational Study. Phys Ther. 2019 Mar 1;99(3):266-275.
48. Czarnecki A, Qiu F, Elbaz-Greener G, Cohen EA, Ko DT, Roifman I, Wijeysundera HC. Variation in Revascularization Practice and Outcomes in Asymptomatic Stable Ischemic Heart Disease. JACC Cardiovasc Interv. 2019 Feb 11;12(3):232-241.
49. Coyle R, Feher M, Jones S, Hamilton M, de Lusignan S. Variation in the diagnosis and control of hypertension is not explained by conventional variables: Cross-sectional database study in English general practice. PLoS One. 2019 Jan 10;14(1):e0210657.
50. O'Sullivan JW, Stevens S, Oke J, Hobbs FDR, Salisbury C, Little P, Goldacre B, Bankhead C, Aronson JK, Heneghan C, Perera R. Practice variation in the use of tests in UK primary care: a retrospective analysis of 16 million tests performed over 3.3 million patient years in 2015/16. BMC Med. 2018 Dec 20;16(1):229.
51. Mendonca SC, Abel GA, Gildea C, McPhail S, Peake MD, Rubin G, Singh H, Hamilton W, Walter FM, Roland MO, Lyratzopoulos G. Associations between general practice characteristics with use of urgent referrals for suspected cancer and endoscopies: a cross-sectional ecological study. Fam Pract. 2019 Oct 8;36(5):573-580.
52. Kooiman LMP, Kamps AWA, Dassel ACM, Brand PLP, Bekhof J. Practice variation among Dutch paediatricians in palivizumab prescription rates: the importance of parental counselling approach. Acta Paediatr. 2019 Jul;108(7):1345-1349.
53. Scheuter C, Wertli MM, Haynes AG, Panczak R, Chiolero A, Perrier A, Rodondi N, Aujesky D. Unwarranted regional variation in vertebroplasty and kyphoplasty in Switzerland: A population-based small area variation analysis. PLoS One. 2018 Dec 10;13(12).
54. Weems L, Strong J, Plummer D, Martin J, Zweng TN, Lindsay J, Paculdo D, Tran M, Peabody J. A Quality Collaboration in Heart Failure and Pneumonia Inpatient Care at Novant Health: Standardizing Hospitalist Practices to Improve Patient Care and System Performance. Jt Comm J Qual Patient Saf. 2019 Mar;45(3):199-206.
55. Sugihara T, Yasunaga H, Matsui H, Kamei J, Fujimura T, Kume H. Regional clinical practice variation in urology: Usage example of the Open Data of the National Database of Health Insurance Claims and Specific Health Checkups of Japan. Int J Urol. 2019 Feb;26(2):303-305.
56. Burgon TB, Cox-Chapman J, Czarnecki C, Kropp R, Guerriere R, Paculdo D, Peabody JW. Engaging Primary Care Providers to Reduce Unwanted Clinical Variation and Support ACO Cost and Quality Goals: A Unique Provider-Payer Collaboration. Popul Health Manag. 2019 Aug;22(4):321-329.
57. Kilinc O, Konya A, Akgun M, Uzaslan E, Sayiner A. A case scenario study for the assessment of physician's behavior in the management of COPD: the WHY study. Int J Chron Obstruct Pulmon Dis. 2018 Sep 5;13:2751-2758.
58. Schulman J, Profit J, Lee HC, Dueñas G, Bennett MV, Parucha J, Jocson MAL, Gould JB. Variations in Neonatal Antibiotic Use. Pediatrics. 2018 Sep;142(3).
59. Sadigh G, Kadom N, Karthik P, Sengupta D, Strauss KJ, Frush D, Applegate KE. Noncontrast Head CT in Children: National Variation in Radiation Dose Indices in the United States. AJNR Am J Neuroradiol. 2018 Aug;39(8):1400-1405.
60. Liao JM, Emanuel EJ, Whittington GL, Small DS, Troxel AB, Zhu J, Zhong W, Navathe AS. Physician practice variation under orthopedic bundled payment. Am J Manag Care. 2018 Jun;24(6):287-293.
61. Zanichelli V, Monnier AA, Gyssens IC, Adriaenssens N, Versporten A, Pulcini C, Le Maréchal M, Tebano G, Vlahovic-Palcevski V, Stanic Benic M, Milanic R, Harbarth S, Hulscher ME, Huttner B. Variation in antibiotic use among and within different settings: a systematic review. J Antimicrob Chemother. 2018 Jun 1;73(suppl_6):vi17-vi29.
62. Huang KZ, Jensen ET, Chen HX, Landes LE, McConnell KA, Almond MA, Johnston DT, Durban R, Jobe L, Frost C, Donnelly S, Antonio B, Safta AM, Quiros JA, Markowitz JE, Dellon ES. Practice Pattern Variation in Pediatric Eosinophilic Esophagitis in the Carolinas EoE Collaborative: A Research Model in Community and Academic Practices. South Med J. 2018 Jun;111(6):328-332.
63. White VanGompel E, Main EK, Tancredi D, Melnikow J. Do provider birth attitudes influence cesarean delivery rate: a cross-sectional study. BMC Pregnancy Childbirth. 2018 May 29;18(1):184.
64. Feufel MA. How to Uncover Sources of Unwarranted Practice Variation: A Case Study in Emergency Medicine. Qual Health Res. 2018 Jul;28(9):1486-1498.
65. Kaiser SV, Garber MD. Using Quality Improvement to Tackle Unwarranted Practice Variation. Hosp Pediatr. 2018 Jun;8(6):375-377.
66. Westert GP, Groenewoud S, Wennberg JE, Gerard C, DaSilva P, Atsma F, Goodman DC. Medical practice variation: public reporting a first necessary step to spark change. Int J Qual Health Care. 2018 Nov 1;30(9):731-735.
67. Gandré C, Gervaix J, Thillard J, Macé JM, Roelandt JL, Chevreul K. Thirty-day Readmission Rates and Associated Factors: A Multilevel Analysis of Practice Variations in French Public Psychiatry. J Ment Health Policy Econ. 2018 Mar 1;21(1):17-28.
68. van Leeuwen MT, Falster MO, Vajdic CM, Crowe PJ, Lujic S, Klaes E, Jorm L, Sedrakyan A. Reoperation after breast-conserving surgery for cancer in Australia: statewide cohort study of linked hospital data. BMJ Open. 2018 Apr 10;8(4):e020858.
69. Glatz AC, Kennedy KF, Rome JJ, O'Byrne ML. Variations in Practice Patterns and Consistency With Published Guidelines for Balloon Aortic and Pulmonary Valvuloplasty: An Analysis of Data From the IMPACT Registry. JACC Cardiovasc Interv. 2018 Mar 26;11(6):529-538.
70. Bucholc M, O'Kane M, Ashe S, Wong-Lin K. Prescriptive variability of drugs by general practitioners. PLoS One. 2018 Feb 20;13(2):e0189599.
71. O'Byrne ML, Kennedy KF, Rome JJ, Glatz AC. Variation in practice patterns in device closure of atrial septal defects and patent ductus arteriosus: An analysis of data from the IMproving Pediatric and Adult Congenital Treatment (IMPACT) registry. Am Heart J. 2018 Feb;196:119-130.
72. Honaker SM, Dugan T, Daftary A, Davis S, Saha C, Baye F, Freeman E, Downs SM. Unexplained Practice Variation in Primary Care Providers' Concern for Pediatric Obstructive Sleep Apnea. Acad Pediatr. 2018 May - Jun;18(4):418-424.
73. Cook DA, Pencille LJ, Dupras DM, Linderbaum JA, Pankratz VS, Wilkinson JM. Practice variation and practice guidelines: Attitudes of generalist and specialist physicians, nurse practitioners, and physician assistants. PLoS One. 2018 Jan 31;13(1).
74. Lubelski D, Alentado VJ, Williams SK, O'Rourke C, Obuchowski NA, Wang JC, Steinmetz MP, Melillo AJ, Benzel EC, Modic MT, Quencer R, Mroz TE. Variability in Surgical Treatment of Spondylolisthesis Among Spine Surgeons. World Neurosurg. 2018 Mar;111:e564-e572.
75. Schuh S, Babl FE, Dalziel SR, Freedman SB, Macias CG, Stephens D, Steele DW, Fernandes RM, Zemek R, Plint AC, Florin TA, Lyttle MD, Johnson DW, Gouin S, Schnadower D, Klassen TP, Bajaj L, Benito J, Kharbanda A, Kuppermann N; Pediatric Emergency Research Networks (PERN). Practice Variation in Acute Bronchiolitis: A Pediatric Emergency Research Networks Study. Pediatrics. 2017 Dec;140(6).
76. Keating NL, Huskamp HA, Schrag D, McWilliams JM, McNeil BJ, Landon BE, Chernew ME, Normand ST. Diffusion of Bevacizumab Across Oncology Practices: An Observational Study. Med Care. 2018 Jan;56(1):69-77.
77. Mori M, Shioda K, Yun JJ, Mangi AA, Darr U, Geirsson A. Pattern and predictors of dual antiplatelet use after coronary artery bypass graft surgery. J Thorac Cardiovasc Surg. 2018 Feb;155(2):632-638.
78. Skeith L, Gonsalves C. Identifying the factors influencing practice variation in thrombosis medicine: A qualitative content analysis of published practice-pattern surveys. Thromb Res. 2017 Nov;159:52-57.
79. Balakrishnan M, Raghavan A, Suresh GK. Eliminating Undesirable Variation in Neonatal Practice: Balancing Standardization and Customization. Clin Perinatol. 2017 Sep;44(3):529-540.
80. Mayer M, Naylor J, Harris I, Badge H, Adie S, Mills K, Descallar J. Evidence base and practice variation in acute care processes for knee and hip arthroplasty surgeries. PLoS One. 2017 Jul 19;12(7).
81. Kristiansen BK, Andersen B, Bro F, Svanholm H, Vedsted P. Impact of GP reminders on follow-up of abnormal cervical cytology: a before-after study in Danish general practice. Br J Gen Pract. 2017 Aug;67(661):e580-e587.
82. Willis TA, West R, Rushforth B, Stokes T, Glidewell L, Carder P, Faulkner S, Foy R; ASPIRE programme team. Variations in achievement of evidence-based, high-impact quality indicators in general practice: An observational study. PLoS One. 2017 Jul 13;12(7).
83. Kadhim-Saleh A, Worrall JC, Taljaard M, Gatien M, Perry JJ. Self-awareness of computed tomography ordering in the emergency department. CJEM. 2018 Mar;20(2):275-283.
84. Hampson LA, Odisho AY, Meng MV, Carroll PR. Variation and Predictors of Surgical Case Costs among Urologists. Urol Pract. 2017 Jul;4(4):277-284.
85. Visscher SJA, van Stel HF. Variation in prevention of child maltreatment by Dutch child healthcare professionals. Child Abuse Negl. 2017 Aug;70:264-273.
86. Samuel SM, Flynn R, Zappitelli M, Dart A, Parekh R, Pinsk M, Mammen C, Wade A, Scott SD; Canadian Childhood Nephrotic Syndrome Project Team*. Factors influencing practice variation in the management of nephrotic syndrome: a qualitative study of pediatric nephrology care providers. CMAJ Open. 2017 Jun 7;5(2):E424-E430.
87. Cook RW, Weiner JA, Schallmo MS, Chun DS, Barth KA, Singh SK, Hsu WK. Effects of Conflicts of Interest on Practice Patterns and Complication Rates in Spine Surgery. Spine (Phila Pa 1976). 2017 Sep 1;42(17):1322-1329.
88. Tavarez MM, Ayers B, Jeong JH, Coombs CM, Thompson A, Hickey RW. Practice Variation and Effects of E-mail-only Performance Feedback on Resource Use in the Emergency Department. Acad Emerg Med. 2017 Aug;24(8):948-956.
89. Eriksen J, Gustafsson LL, Ateva K, Bastholm-Rahmner P, Ovesjö ML, Jirlow M, Juhasz-Haverinen M, Lärfars G, Malmström RE, Wettermark B, Andersén-Karlsson E; Stockholm DTC. High adherence to the 'Wise List' treatment recommendations in Stockholm: a 15-year retrospective review of a multifaceted approach promoting rational use of medicines. BMJ Open. 2017 May 2;7(4).
90. Duell D, Koolman X, Portrait F. Practice variation in the Dutch long-term care and the role of supply-sensitive care: Is access to the Dutch long-term care equitable? Health Econ. 2017 Dec;26(12):1728-1742.
91. Lieu TA, Ray GT, Prausnitz SR, Habel LA, Alexeeff S, Li Y, Ramsey SD, Phelps CE, Chawla N, C O'Neill S, Mandelblatt JS. Oncologist and organizational factors associated with variation in breast cancer multigene testing. Breast Cancer Res Treat. 2017 May;163(1):167-176.
92. Sinnige J, Braspenning JC, Schellevis FG, Hek K, Stirbu I, Westert GP, Korevaar JC. [Inter-practice variation in polypharmacy prevalence amongst older patients in primary care]. Ned Tijdschr Geneeskd. 2017;161:D864.
93. Ren CL, Schechter MS. Reducing practice variation through clinical pathways-Is it enough? Pediatr Pulmonol. 2017 May;52(5):577-579.
94. Pahle AS, Sørli D, Kristiansen IS, Deraas TS, Halvorsen PA. Practice variation in surgical procedures and IUD-insertions among general practitioners in Norway - a longitudinal study. BMC Fam Pract. 2017 Jan 21;18(1):7.
95. Mendlovic J, Gordon ES, Haklai Z, Meron J, Afek A. Geographic variation in selected hospital procedures and services in the Israeli health care system. Isr J Health Policy Res. 2017 Jan 16;6:4.
96. Fullerton K, Depinet H, Iyer S, Hall M, Herr S, Morton I, Lee T, Melzer-Lange M. Association of Hospital Resources and Imaging Choice for Appendicitis in Pediatric Emergency Departments. Acad Emerg Med. 2017 Apr;24(4):400-409.
97. Vankan E, Schoorel EN, van Kuijk SM, Mol BJ, Nijhuis JG, Aardenburg R, Alink M, de Boer K, Delemarre FM, Dirksen CD, van Dooren IM, Franssen MT, Kaplan M, Kleiverda G, Kuppens SM, Kwee A, Langenveld J, Lim FT, Melman S, Sikkema MJ, Smits LJ, Visser H, Woiski M, Scheepers HC, Hermens RP. Practice variation of vaginal birth after cesarean and the influence of risk factors at patient level: a retrospective cohort study. Acta Obstet Gynecol Scand. 2017 Feb;96(2):158-165.
98. Duggan A, Koff E, Marshall V. Clinical variation: why it matters. Med J Aust. 2016 Nov 21;205(10):S3-S4.
99. Andrews SE, Allshouse AA, Moore GS, Alston MJ, Metz TD. Influence of Residency Training on Operative Vaginal Delivery in Independent Practice. J Reprod Med. 2016 Nov-Dec;61(11-12):562-8.
100. Dart AB, Zappitelli M, Sood MM, Alexander RT, Arora S, Erickson RL, Kroeker K, Soo A, Manns BJ, Samuel SM. Variation in estimated glomerular filtration rate at dialysis initiation in children. Pediatr Nephrol. 2017 Feb;32(2):331-340.
101. Kini V, Weiner RB, McCarthy FH, Wiegers SE, Kirkpatrick JN. Association of Liability Concerns with Decisions to Order Echocardiography and Cardiac Stress Tests with Imaging. J Am Soc Echocardiogr. 2016 Dec;29(12):1155-1160.
102. Alapati D, Jassar R, Shaffer TH. Management of Supplemental Oxygen for Infants with Persistent Pulmonary Hypertension of Newborn: A Survey. Am J Perinatol. 2017 Feb;34(3):276-282.
103. Yoshida K, Krille L, Dreger S, Hoenig L, Merzenich H, Yasui K, Kumagai A, Ohtsuru A, Uetani M, Mildenberger P, Takamura N, Yamashita S, Zeeb H, Kudo T. Pediatric computed tomography practice in Japanese university hospitals from 2008-2010: did it differ from German practice? J Radiat Res. 2017 Jan;58(1):135-141.
104. van Essen TA, de Ruiter GC, Kho KH, Peul WC. Neurosurgical Treatment Variation of Traumatic Brain Injury: Evaluation of Acute Subdural Hematoma Management in Belgium and The Netherlands. J Neurotrauma. 2017 Feb 15;34(4):881-889.
105. Haastrup PF, Rasmussen S, Hansen JM, Christensen RD, Søndergaard J, Jarbøl DE. General practice variation when initiating long-term prescribing of proton pump inhibitors: a nationwide cohort study. BMC Fam Pract. 2016 May 28;17:57.
106. Brabers AE, van Dijk L, Groenewegen PP, van Peperstraten AM, de Jong JD. Does a strategy to promote shared decision-making reduce medical practice variation in the choice of either single or double embryo transfer after in vitro fertilisation? A secondary analysis of a randomised controlled trial. BMJ Open. 2016 May 6;6(5):e010894.
107. Sinnige J, Braspenning JC, Schellevis FG, Hek K, Stirbu I, Westert GP, Korevaar JC. Inter-practice variation in polypharmacy prevalence amongst older patients in primary care. Pharmacoepidemiol Drug Saf. 2016 Sep;25(9):1033-41.
108. Aliaga S, Zhang J, Long DL, Herring AH, Laughon M, Boggess K, Reddy UM, Grantz KL. Center Variation in the Delivery of Indicated Late Preterm Births. Am J Perinatol. 2016 Aug;33(10):1008-16.
109. Dalton JE, Zidar DA, Udeh BL, Patel MR, Schold JD, Dawson NV. Practice Variation Among Hospitals in Revascularization Therapy and Its Association With Procedure-related Mortality. Med Care. 2016 Jun;54(6):623-31.
110. Guerrier K, Shamszad P, Czosek RJ, Spar DS, Knilans TK, Anderson JB. Variation in Antiarrhythmic Management of Infants Hospitalized with Supraventricular Tachycardia: A Multi-Institutional Analysis. Pediatr Cardiol. 2016 Jun;37(5):946-52.
111. Fantini G, Tibaldi G, Rucci P, Gibertoni D, Vezzoli M, Cifarelli L, Tiraferri R, Munizza C. Quality of care indicators for schizophrenia: determinants of observed variations among Italian Departments of Mental Health. Results from the ETAS DSM study. Epidemiol Psychiatr Sci. 2017 Jun;26(3):299-313.
112. Clough JD, Rajkumar R, Crim MT, Ott LS, Desai NR, Conway PH, Maresh S, Kahvecioglu DC, Krumholz HM. Practice-Level Variation in Outpatient Cardiac Care and Association With Outcomes. J Am Heart Assoc. 2016 Feb 23;5(2).
113. Groeneveld IF, Meesters JJ, Arwert HJ, Roux-Otter N, Ribbers GM, van Bennekom CA, Goossens PH, Vliet Vlieland TP. Practice variation in the structure of stroke rehabilitation in four rehabilitation centres in the Netherlands. J Rehabil Med. 2016 Mar;48(3):287-92.
114. Wasfy JH, Kennedy KF, Chen JS, Ferris TG, Maddox TM, Yeh RW. Practice Variation in Triple Therapy for Patients With Both Atrial Fibrillation and Coronary Artery Disease: Insights From the ACC's National Cardiovascular Data Registry. JACC Clin Electrophysiol. 2016 Feb;2(1):36-43.
115. Cameron DB, Rangel SJ. Variation in pediatric surgical care. Semin Pediatr Surg. 2015 Dec;24(6):291-4.
116. Djulbegovic B, Hamm RM, Mayrhofer T, Hozo I, Van den Ende J. Rationality, practice variation and person-centred health policy: a threshold hypothesis. J Eval Clin Pract. 2015 Dec;21(6):1121-4.
117. Bertille N, Pons G, Khoshnood B, Fournier-Charrière E, Chalumeau M. Symptomatic Management of Fever in Children: A National Survey of Healthcare Professionals' Practices in France. PLoS One. 2015 Nov 23;10(11).
118. Loeffen EA, Mulder RL, van de Wetering MD, Font-Gonzalez A, Abbink FC, Ball LM, Loeffen JL, Michiels EM, Segers H, Kremer LC, Tissing WJ. Current variations in childhood cancer supportive care in the Netherlands. Cancer. 2016 Feb 15;122(4):642-50.
119. Godard S, Herry C, Westergaard P, Scales N, Brown S, Burns K, Mehta S, Jacono F, Kubelik D, Maziak DE, Marshall J, Martin C, Seely A. Practice variation in spontaneous breathing trial performance and documentation. Can Respir J. 2015 Nov 17.
120. Teunis T, Janssen SJ, Guitton TG, Vranceanu AM, Goos B, Ring D. Surgeon personality is associated with recommendation for operative treatment. Hand (NY). 2015 Dec;10(4):779-84.
121. Krska J, du Plessis R, Chellaswamy H. Implementation of NHS Health Checks in general practice: variation in delivery between practices and practitioners. Prim Health Care Res Dev. 2016 Jul;17(4):385-92.
122. Beeler PE, Orav EJ, Seger DL, Dykes PC, Bates DW. Provider variation in responses to warnings: do the same providers run stop signs repeatedly? J Am Med Inform Assoc. 2016 Apr;23(e1):e93-8.
123. Gabriel RA, Lemay A, Beutler SS, Dutton RP, Urman RD. Practice Variations in Anesthesia for Carotid Endarterectomies and Associated Outcomes. J Cardiothorac Vasc Anesth. 2016 Jan;30(1):23-9.
124. Gabriel RA, Kaye AD, Jones MR, Dutton RP, Urman RD. Practice Variations in Anesthetic Care and Its Effect on Clinical Outcomes for Primary Total Hip Arthroplasties. J Arthroplasty. 2016 Apr;31(4):918-22.
125. Weinberg JG, Evans FJ, Burns KM, Pearson GD, Kaltman JR. Surgical ligation of patent ductus arteriosus in premature infants: trends and practice variation. Cardiol Young. 2016 Aug;26(6):1107-14.
126. Goldstein BH, Holzer RJ, Trucco SM, Porras D, Murphy J, Foerster SR, El-Said HG, Beekman RH 3rd, Bergersen L. Practice Variation in Single-Ventricle Patients Undergoing Elective Cardiac Catheterization: A Report from the Congenital Cardiac Catheterization Project on Outcomes (C3PO). Congenit Heart Dis. 2016 Mar-Apr;11(2):122-35.
127. Nippita TA, Trevena JA, Patterson JA, Ford JB, Morris JM, Roberts CL. Variation in hospital rates of induction of labour: a population-based record linkage study. BMJ Open. 2015 Sep 2;5(9):e008755.
128. Busby J, Purdy S, Hollingworth W. A systematic review of the magnitude and cause of geographic variation in unplanned hospital admission rates and length of stay for ambulatory care sensitive conditions. BMC Health Serv Res. 2015 Aug 13;15:324.
129. Tan A, Zhou J, Kuo YF, Goodwin JS. Variation among Primary Care Physicians in the Use of Imaging for Older Patients with Acute Low Back Pain. J Gen Intern Med. 2016 Feb;31(2):156-163.
130. van IJsselmuiden MN, Detollenaere RJ, Kampen MY, Engberts MK, van Eijndhoven HW. Practice pattern variation in surgical management of pelvic organ prolapse and urinary incontinence in The Netherlands. Int Urogynecol J. 2015 Nov;26(11):1649-56.
131. Apramian T, Watling C, Lingard L, Cristancho S. Adaptation and innovation: a grounded theory study of procedural variation in the academic surgical workplace. J Eval Clin Pract. 2015 ct;21(5):911-8.
132. Velzel J, Roovers JP, Van der Vaart CH, Broekman B, Vollebregt A, Hakvoort R. A nationwide survey concerning practices in pessary use for pelvic organ prolapse in The Netherlands: identifying needs for further research. Int Urogynecol J. 2015 Oct;26(10):1453-8.
133. Douven R, Mocking R, Mosca I. The effect of physician remuneration on regional variation in hospital treatments. Int J Health Econ Manag. 2015 Jun;15(2):215-240.
134. Lim MY, Buckner TW, Kasthuri RS, Ma AD, Key NS. Management of adult non-severe haemophilia A patients with inhibitors: a practice-pattern survey. Haemophilia. 2015 Sep;21(5).
135. Göpffarth D, Kopetsch T, Schmitz H. Determinants of Regional Variation in Health Expenditures in Germany. Health Econ. 2016 Jul;25(7):801-15.
136. Day LW, Nazareth M, Sewell JL, Williams JL, Lieberman DA. Practice variation in PEG tube placement: trends and predictors among providers in the United States. Gastrointest Endosc. 2015 Jul;82(1):37-45.
137. Gupta P, Tang X, Gossett JM, Gall CM, Lauer C, Rice TB, Wetzel RC. Variation of ventilation practices with center volume after pediatric heart surgery. Clin Cardiol. 2015 Mar;38(3):178-84.
138. Aronson PL, Thurm C, Williams DJ, Nigrovic LE, Alpern ER, Tieder JS, Shah SS, McCulloh RJ, Balamuth F, Schondelmeyer AC, Alessandrini EA, Browning WL, Myers AL, Neuman MI; Febrile Young Infant Research Collaborative. Association of clinical practice guidelines with emergency department management of febrile infants ≤56 days of age. J Hosp Med. 2015 Jun;10(6):358-65.
139. Djulbegovic B, van den Ende J, Hamm RM, Mayrhofer T, Hozo I, Pauker SG; International Threshold Working Group (ITWG). When is rational to order a diagnostic test, or prescribe treatment: the threshold model as an explanation of practice variation. Eur J Clin Invest. 2015 May;45(5):485-93.
140. Offerhaus PM, Otten W, Boxem-Tiemessen JC, de Jonge A, van der Pal-de Bruin KM, Scheepers PL, Lagro-Janssen AL. Variation in intrapartum referral rates in primary midwifery care in the Netherlands: a discrete choice experiment. Midwifery. 2015 Apr;31(4):e69-78.
141. Burton C, Cameron I, Anderson N. Explaining the variation between practices in the duration of new antidepressant treatment: a database cohort study in primary care. Br J Gen Pract. 2015 Feb;65(631):e114-20.
142. Lagares A, Munarriz PM, Ibáñez J, Arikán F, Sarabia R, Morera J, Gabarrós A, Horcajadas Á; Grupo de Patología Vascular de la SENEC. [Variability in the management of aneurysmal subarachnoid haemorrhage in Spain: Analysis of the prospective multicenter database from the Working Group on Neurovascular Diseases of the Spanish Society of Neurosurgery]. Neurocirugia (Astur). 2015 Jul-Aug;26(4):167-79.
143. Tomlin AM, Gillies TD, Tilyard MW, Dovey SM. Variation in the pharmaceutical costs of New Zealand general practices: a national database linkage study. J Public Health (Oxf). 2016 Mar;38(1):138-46.
144. Verschoor MA, Lemmers M, Wekker MZ, Huirne JA, Goddijn M, Mol BW, Ankum WM. Practice variation in the management of first trimester miscarriage in the Netherlands: a nationwide survey. Obstet Gynecol Int. 2014;2014:387860.
145. Ananthakrishnan AN, Kwon J, Raffals L, Sands B, Stenson WF, McGovern D, Kwon JH, Rheaume RL, Sandler RS. Variation in treatment of patients with inflammatory bowel diseases at major referral centers in the United States. Clin Gastroenterol Hepatol. 2015 Jun;13(6):1197-200.
146. Bohossian HB, Park AW, Holcroft C. The impact of individual variation analysis on myocardial perfusion imaging utilization within a hospitalist group. J Hosp Med. 2015 Mar;10(3):190-3.
147. Stanley RM, Hoyle JD Jr, Dayan PS, Atabaki S, Lee L, Lillis K, Gorelick MH, Holubkov R, Miskin M, Holmes JF, Dean JM, Kuppermann N; Pediatric Emergency Care Applied Research Network (PECARN). Emergency department practice variation in computed tomography use for children with minor blunt head trauma. J Pediatr. 2014 Dec;165(6):1201-1206.
148. Fierro JL, Prasad PA, Localio AR, Grundmeier RW, Wasserman RC, Zaoutis TE, Gerber JS. Variability in the diagnosis and treatment of group a streptococcal pharyngitis by primary care pediatricians. Infect Control Hosp Epidemiol. 2014 Oct;35 Suppl 3:S79-85.
149. Patel HD, Humphreys E, Trock BJ, Han M, Carter HB. Practice patterns and individual variability of surgeons performing radical prostatectomy at a high volume academic center. J Urol. 2015 Mar;193(3):812-9.
150. Patterson JA, Roberts CL, Isbister JP, Irving DO, Nicholl MC, Morris JM, Ford JB. What factors contribute to hospital variation in obstetric transfusion rates? Vox Sang. 2015 Jan;108(1):37-45.
151. Florin TA, Byczkowski T, Ruddy RM, Zorc JJ, Test M, Shah SS. Variation in the management of infants hospitalized for bronchiolitis persists after the 2006 American Academy of Pediatrics bronchiolitis guidelines. J Pediatr. 2014 Oct;165(4):786-92.
152. Berlin C, Busato A, Rosemann T, Djalali S, Maessen M. Avoidable hospitalizations in Switzerland: a small area analysis on regional variation, density of physicians, hospital supply and rurality. BMC Health Serv Res. 2014 Jul 3;14:289.
153. Weeks JC, Uno H, Taback N, Ting G, Cronin A, D'Amico TA, Friedberg JW, Schrag D. Interinstitutional variation in management decisions for treatment of 4 common types of cancer: A multi-institutional cohort study. Ann Intern Med. 2014 Jul 1;161(1):20-30.
154. Sandiford P, Zhou L, Salvetto M, Johnson LF. Measuring unexplained variation in acute hospital use by patients enrolled with northern New Zealand general practices. J Prim Health Care. 2014 Jun 1;6(2):93-100.
155. Mangham-Jefferies L, Hanson K, Mbacham W, Onwujekwe O, Wiseman V. What determines providers' stated preference for the treatment of uncomplicated malaria? Soc Sci Med. 2014 Mar;104:98-106.
156. Farias M, Rathod RH. Standardized clinical assessment and management plans: a clinician-led approach to unwarranted practice variation. Virtual Mentor. 2014 Feb 1;16(2):115-9.
157. Nguyen YL, Perrodeau E, Guidet B, Trinquart L, Richard JC, Mercat A, Jolliet P, Ravaud P, Brochard L; REVA network. Mechanical ventilation and clinical practice heterogeneity in intensive care units: a multicenter case-vignette study. Ann Intensive Care. 2014 Feb 1;4(1):2.
158. Melchior H, Schulz H, Härter M. [Significance of regional variations in the prevalence and treatment of depressive disorders and implications for health-care research]. undesgesundheitsblatt Gesundheitsforschung Gesundheitsschutz. 2014 Feb;57(2):224-33.
159. Stock S, Danner M. [Does the elicitation of stakeholder attitudes and preferences add valuable information to small-area analyses? A health policy perspective]. Bundesgesundheitsblatt Gesundheitsforschung Gesundheitsschutz. 2014 Feb;57(2):188-96.
160. Swart E, Graf von Stillfried D, Koch-Gromus U. [Small-area health-care research: where science, practice, and policy meet]. Bundesgesundheitsblatt Gesundheitsforschung Gesundheitsschutz. 2014 Feb;57(2):161-3.
161. Corallo AN, Croxford R, Goodman DC, Bryan EL, Srivastava D, Stukel TA. A systematic review of medical practice variation in OECD countries. Health Policy. 2014 Jan;114(1):5-14.
162. Lee YY, Roberts CL, Patterson JA, Simpson JM, Nicholl MC, Morris JM, Ford JB. Unexplained variation in hospital caesarean section rates. Med J Aust. 2013 Sep 2;199(5):348-53.
163. Cook DJ, Thompson JE, Suri R, Prinsen SK. Surgical Process Improvement: Impact of a Standardized Care Model With Electronic Decision Support to Improve Compliance With SCIP Inf-9. Am J Med Qual. 2014 Jul-Aug;29(4):323-8.
164. Dobloug A, Grytten J, Holst D. Dentist-specific variation in diagnosis of caries - a multilevel analysis. Community Dent Oral Epidemiol. 2014 Apr;42(2):185-91.
165. Koefoed MM, Søndergaard J, Christensen Rd, Jarbøl DE. General practice variation in spirometry testing among patients receiving first-time prescriptions for medication targeting obstructive lung disease in Denmark: a population-based observational study. BMC Fam Pract. 2013 Aug 7;14:113.
166. Florin TA, French B, Zorc JJ, Alpern ER, Shah SS. Variation in emergency department diagnostic testing and disposition outcomes in pneumonia. Pediatrics. 2013 Aug;132(2):237-44.
167. Henke RM, Maeda JL, Marder WD, Friedman BS, Wong HS. Medicare and commercial inpatient resource use: impact of hospital competition. Am J Manag Care. 2013 Jun 1;19(6):e238-48.
168. Olson DM, Lewis LS, Bader MK, Bautista C, Malloy R, Riemen KE, McNett MM. Significant practice pattern variations associated with intracranial pressure monitoring. J Neurosci Nurs. 2013 Aug;45(4):186-93.
169. Mulley AG Jr. The global role of health care delivery science: learning from variation to build health systems that avoid waste and harm. J Gen Intern Med. 2013 Sep;28 Suppl 3:S646-53.
170. Milgrom P, Huebner CE, Mancl LA, Chi DL, Garson G, Grembowski D. County-level characteristics as predictors of dentists' ECC counseling in the USA: a survey study. BMC Oral Health. 2013 May 20;13:23.
171. Graverholt B, Riise T, Jamtvedt G, Husebo BS, Nortvedt MW. Acute hospital admissions from nursing homes: predictors of unwarranted variation? Scand J Public Health. 2013 Jun;41(4):359-65.
172. Tomson CR, van der Veer SN. Learning from practice variation to improve the quality of care. Clin Med (Lond). 2013 Feb;13(1):19-23.
173. Locher JL, Bonner JA, Carroll WR, Caudell JJ, Allison JJ, Kilgore ML, Ritchie CS, Tajeu GS, Yuan Y, Roth DL. Patterns of prophylactic gastrostomy tube placement in head and neck cancer patients: a consideration of the significance of social support and practice variation. Laryngoscope. 2013 Aug;123(8):1918-25.
174. Ghaffarzadegan N, Epstein AJ, Martin EG. Practice variation, bias, and experiential learning in cesarean delivery: a data-based system dynamics approach. Health Serv Res. 2013 Apr;48(2 Pt 2):713-34.
175. Rodríguez-Martínez P, Peiró S, Librero J, Bernal-Delgado E, Gisbert-Grifo M, Calabuig-Pérez J, Ridao-López M, Sanfélix-Gimeno G. Carpal tunnel release surgery: small-area variation and impact of ambulatory surgery in the autonomous region of Valencia, Spain. Gac Sanit. 2013 May-Jun;27(3):214-9.
176. Lay-Yee R, Scott A, Davis P. Patterns of family doctor decision making in practice context. What are the implications for medical practice variation and social disparities? Soc Sci Med. 2013 Jan;76(1):47-56.
177. Verghese GR, Friedman KG, Rathod RH, Meiri A, Saleeb SF, Graham DA, Geggel RL, Fulton DR. Resource Utilization Reduction for Evaluation of Chest Pain in Pediatrics Using a Novel Standardized Clinical Assessment and Management Plan (SCAMP). J Am Heart Assoc. 2012 Apr;1(2).
178. Albrecht E, Taffe P, Yersin B, Schoettker P, Decosterd I, Hugli O. Undertreatment of acute pain (oligoanalgesia) and medical practice variation in prehospital analgesia of adult trauma patients: a 10 yr retrospective study. Br J Anaesth. 2013 Jan;110(1):96-106.
179. Peiró S, Bernal-Delgado E. [Médical practice variation: supporting the null hypothesis in turbulent times]. Rev Esp Salud Publica. 2012 May-Jun;86(3):213-7.
180. Patrick SW, Schumacher RE, Davis MM. Variation in lumbar punctures for early onset neonatal sepsis: a nationally representative serial cross-sectional analysis, 2003-2009. BMC Pediatr. 2012 Aug 28;12:134.
181. Jain NB, Kuye I, Higgins LD, Warner JJ. Surgeon volume is associated with cost and variation in surgical treatment of proximal humeral fractures. Clin Orthop Relat Res. 2013 Feb;471(2):655-64.
182. Basu J, Thumula V, Mobley LR. Changes in preventable hospitalization patterns among the adults: a small area analysis of US states. J Ambul Care Manage. 2012 Jul-Sep;35(3):226-37.
183. So C, Kirby KA, Mehta K, Hoffman RM, Powell AA, Freedland SJ, Sirovich B, Yano EM, Walter LC. Medical center characteristics associated with PSA screening in elderly veterans with limited life expectancy. J Gen Intern Med. 2012 Jun;27(6):653-60.
184. Busato A, Matter P, Künzi B, Goodman D. Geographic variation in the cost of ambulatory care in Switzerland. J Health Serv Res Policy. 2012 Jan;17(1):18-23.
185. Shwartz M, Peköz EA, Labonte A, Heineke J, Restuccia JD. Bringing responsibility for small area variations in hospitalization rates back to the hospital: the propensity to hospitalize index and a test of the Roemer's Law. Med Care. 2011 Dec;49(12):1062-7.
186. Pourat N, Marcus M. Variations in self-reported provision of services by general dentists in private practice. J Am Dent Assoc. 2011 Sep;142(9):1050-60.
187. Haynes K, Bilker WB, Tenhave TR, Strom BL, Lewis JD. Temporal and within practice variability in the health improvement network. Pharmacoepidemiol Drug Saf. 2011 Sep;20(9):948-55.
188. Ohlsson H, Vervloet M, van Dijk L. Practice variation in a longitudinal perspective: a multilevel analysis of the prescription of simvastatin in general practices between 2003 and 2009. Eur J Clin Pharmacol. 2011 Dec;67(12):1205-11.
189. Scales CD Jr, Krupski TL, Curtis LH, Matlaga B, Lotan Y, Pearle MS, Saigal C, Preminger GM; Urologic Diseases in America Project. Practice variation in the surgical management of urinary lithiasis. J Urol. 2011 Jul;186(1):146-50.
190. Kim AS, Sidney S, Klingman JG, Johnston SC. Practice variation in neuroimaging to evaluate dizziness in the ED. Am J Emerg Med. 2012 Jun;30(5):665-72.
191. Mercuri M, Gafni A. Medical practice variations: what the literature tells us (or does not) about what are warranted and unwarranted variations. J Eval Clin Pract. 2011 Aug;17(4):671-7.
192. Dudley N. Tackling practice variation. Threats and opportunities of the NHS reforms. BMJ. 2011 Apr 8;342:d2269.
193. Forman DE. Nursing homes and the care of heart failure residents: what have we learned? J Am Med Dir Assoc. 2011 Oct;12(8):544.
194. Venkatraman G, Likosky DS, Morrison D, Zhou W, Finlayson SR, Goodman DC. Small area variation in endoscopic sinus surgery rates among the Medicare population. Arch Otolaryngol Head Neck Surg. 2011 Mar;137(3):253-7.
195. Westert GP, Faber M. Commentary: the Dutch approach to unwarranted medical practice variation. BMJ. 2011 Mar 17;342:d1429.
196. Ip IK, Mortele KJ, Prevedello LM, Khorasani R. Focal cystic pancreatic lesions: assessing variation in radiologists' management recommendations. Radiology. 2011 Apr;259(1):136-41.
197. Freedman SB, Gouin S, Bhatt M, Black KJ, Johnson D, Guimont C, Joubert G, Porter R, Doan Q, van Wylick R, Schuh S, Atenafu E, Eltorky M, Cho D, Plint A; Pediatric Emergency Research Canada. Prospective assessment of practice pattern variations in the treatment of pediatric gastroenteritis. Pediatrics. 2011 Feb;127(2):e287-95.
198. Morrato EH, Druss BG, Hartung DM, Valuck RJ, Thomas D, Allen R, Campagna E, Newcomer JW. Small area variation and geographic and patient-specific determinants of metabolic testing in antipsychotic users. Pharmacoepidemiol Drug Saf. 2011 Jan;20(1):66-75.
199. Mercuri M, Natarajan MK, Norman G, Gafni A. An even smaller area variation: differing practice patterns among interventional cardiologists within a single high volume tertiary cardiac centre. Health Policy. 2012 Feb;104(2):179-85.
200. Gellhorn AC, Chan L, Martin B, Friedly J. Management patterns in acute low back pain: the role of physical therapy. Spine (Phila Pa 1976). 2012 Apr 20;37(9):775-82.
201. Kennedy PJ, Leathley CM, Hughes CF. Clinical practice variation. Med J Aust. 2010 Oct 18;193(S8):S97-9.
202. Neovius M, Sundström A, Simard J, Wettermark B, Cars T, Feltelius N, Askling J, Klareskog L; ARTIS Study Group. Small-area variations in sales of TNF inhibitors in Sweden between 2000 and 2009. Scand J Rheumatol. 2011 Jan;40(1):8-15.
203. Gauld R, Horwitt J, Williams S, Cohen AB. What Strategies do US hospitals employ to reduce unwarranted clinical practice variations? Am J Med Qual. 2011 Mar-Apr;26(2):120-6.
204. Vægter K, Wahlström R, Wedel H, Svärdsudd K. Effect of mailed feedback on drug prescribing profiles in general practice: a seven-year longitudinal study in Storstrøm County, Denmark. Ups J Med Sci. 2010 Nov;115(4):238-44.
205. Barnett R, Malcolm L. GP practice variation in hospitalisation rates: a study of partnership health-enrolled patients. J Prim Health Care. 2010 Jun;2(2):111-7.
206. Dalemo S, Hjerpe P, Ohlsson H, Eggertsen R, Merlo J, Boström KB. Variation in plasma calcium analysis in primary care in Sweden--a multilevel analysis. BMC Fam Pract. 2010 May 30;11:43.
207. Yuen J, Clark A, Ng JQ, Morlet N, Keeffe J, Taylor HR, Preen DB. Further survey of Australian ophthalmologist's diabetic retinopathy management: did practice adhere to National Health and Medical Research Council guidelines? Clin Exp Ophthalmol. 2010 Aug;38(6):613-9.
208. Kuwabara K, Matsuda S, Fushimi K, Ishikawa KB, Horiguchi H, Fujimori K. Variations in the preoperative resources use and the practice pattern in Japanese cholecystectomy patients. Surg Today. 2010 Apr;40(4):334-46.
209. Hubbard Winkler SL, Cowper Ripley DC, Wu S, Reker DM, Vogel B, Fitzgerald SG, Mann WC, Hoenig H. Demographic and clinical variation in Veterans Health Administration provision of assistive technology devices to veterans poststroke. Arch Phys Med Rehabil. 2010 Mar;91(3):369-377.
210. Aakvik A, Holmås TH, Kamrul Islam M. Does variation in general practitioner (GP) practice matter for the length of sick leave? A multilevel analysis based on Norwegian GP-patient data. Soc Sci Med. 2010 May;70(10):1590-8.
211. Pefoyo AJ, Laurier C, Rivard M. [Determinants of geographic variations in asthma medication in Quebec]. Rev Mal Respir. 2010;27(1):49-62.
212. Mousquès J, Renaud T, Scemama O. Is the "practice style" hypothesis relevant for general practitioners? An analysis of antibiotics prescription for acute rhinopharyngitis. Soc Sci Med. 2010 Apr;70(8):1176-84.
213. Billington EO, Zygun DA, Stelfox HT, Peets AD. Intensivists' base specialty of training is associated with variations in mortality and practice patterns. Crit Care. 2009;13(6):R209.
214. de Jong JD, Groenewegen PP, Spreeuwenberg P, Schellevis F, Westert GP. Do guidelines create uniformity in medical practice? Soc Sci Med. 2010 Jan;70(2):209-16.
215. Hampers LC. Practice variation with febrile infants: delight in disorder? Pediatrics. 2009 Aug;124(2):783-5.
216. Lutfey KE, Link CL, Marceau LD, Grant RW, Adams A, Arber S, Siegrist J, Bönte M, von dem Knesebeck O, McKinlay JB. Diagnostic certainty as a source of medical practice variation in coronary heart disease: results from a cross-national experiment of clinical decision making. Med Decis Making. 2009 Sep-Oct;29(5):606-18.
217. Schneiderman R, Kirkby S, Turenne W, Greenspan J. Incubator weaning in preterm infants and associated practice variation. J Perinatol. 2009 Aug;29(8):570-4.
218. Nielen MM, Schellevis FG, Verheij RA. Inter-practice variation in diagnosing hypertension and diabetes mellitus: a cross-sectional study in general practice. BMC Fam Pract. 2009 Jan 21;10:6.
219. Baker RA, Newland RF. Continous quality improvement of perfusion practice: the role of electronic data collection and statistical control charts. Perfusion. 2008 Jan;23(1):7-16.
220. Landrigan CP, Conway PH, Stucky ER, Chiang VW, Ottolini MC. Variation in pediatric hospitalists' use of proven and unproven therapies: a study from the Pediatric Research in Inpatient Settings (PRIS) network. J Hosp Med. 2008 Jul;3(4):292-8.
221. Brown HS 3rd. Lawsuit activity, defensive medicine, and small area variation: the case of Cesarean sections revisited. Health Econ Policy Law. 2007 Jul;2(Pt 3):285-96.
222. Parente ST, Phelps CE, O'Connor PJ. Economic analysis of medical practice variation between 1991 and 2000: the impact of patient outcomes research teams (PORTs). Int J Technol Assess Health Care. 2008 Summer;24(3):282-93.
223. Altschuler A, Collins B, Lewis JD, Velayos F, Allison JE, Hutfless S, Liu L, Herrinton LJ. Gastroenterologists' attitudes and self-reported practices regarding inflammatory bowel disease. Inflamm Bowel Dis. 2008 Jul;14(7):992-9.
224. Landrum MB, Meara ER, Chandra A, Guadagnoli E, Keating NL. Is spending more always wasteful? The appropriateness of care and outcomes among colorectal cancer patients. Health Aff (Millwood). 2008 Jan-Feb;27(1):159-68.
225. Barnato AE, Bost JE, Farrell MH, Lave JR, Arnold RM, Rubio DM, Angus DC. Relationship between staff perceptions of hospital norms and hospital-level end-of-life treatment intensity. J Palliat Med. 2007 Oct;10(5):1093-100.
226. Poitras S, Blais R, Swaine B, Rossignol M. Practice patterns of physiotherapists in the treatment of work-related back pain. J Eval Clin Pract. 2007 Jun;13(3):412-21.
227. Ohlsson H, Merlo J. Understanding the effects of a decentralized budget on physicians' compliance with guidelines for statin prescription--a multilevel methodological approach. BMC Health Serv Res. 2007 May 8;7:68.
228. Baicker K, Buckles KS, Chandra A. Geographic variation in the appropriate use of cesarean delivery. Health Aff (Millwood). 2006 Sep-Oct;25(5):w355-67.
229. Vanobbergen JN, De Visschere LM. Factors contributing to the variation in oral hygiene practices and facilities in long-term care institutions for the elderly. Community Dent Health. 2005 Dec;22(4):260-5.
230. Ohlsson H, Lindblad U, Lithman T, Ericsson B, Gerdtham UG, Melander A, Råstam L, Merlo J. Understanding adherence to official guidelines on statin prescribing in primary health care--a multi-level methodological approach. Eur J Clin Pharmacol. 2005 Oct;61(9):657-65.
231. Greenberg GA, Rosenheck RA. Does system reform reduce geographic variation in mental health system performance. Psychiatr Q. 2005 Fall;76(3):231-42.
232. Burns KE, Sinuff T, Adhikari NK, Meade MO, Heels-Ansdell D, Martin CM, Cook DJ. Bilevel noninvasive positive pressure ventilation for acute respiratory failure: survey of Ontario practice. Crit Care Med. 2005 Jul;33(7):1477-83.
233. Love T, Crampton P, Salmond C, Dowell A. Patterns of medical practice variation: variability in referral for back pain by New Zealand general practitioners. N Z Med J. 2005 Apr 1;118(1212).
234. Jonkman JN, McCarty D, Harwood HJ, Normand SL, Caspi Y. Practice variation and length of stay in alcohol and drug detoxification centers. J Subst Abuse Treat. 2005 Jan;28(1):11-8.
235. Wennberg JE. Practice variation: implications for our health care system. Manag Care. 2004 Sep;13(9 Suppl):3-7.
236. Wennberg JE. Practice variations and health care reform: connecting the dots. Health Aff (Millwood). 2004;Suppl Variation:VAR140-4.
237. Sekimoto M, Imanaka Y, Evans E, Ishizaki T, Hirose M, Hayashida K, Fukui T. Practice variation in perioperative antibiotic use in Japan. Int J Qual Health Care. 2004 Oct;16(5):367-73.
238. Westert GP, Groenewegen PP, Boshuizen HC, Spreeuwenberg PM, Steultjens MP. Medical practice variations in hospital care; time trends of a spatial phenomenon. Health Place. 2004 Sep;10(3):215-20.
239. Margo CE. Quality care and practice variation: the roles of practice guidelines and public profiles. Surv Ophthalmol. 2004 May-Jun;49(3):359-71. Review.
240. Almog M, Curtis S, Copeland A, Congdon P. Geographical variation in acute psychiatric admissions within New York City 1990-2000: growing inequalities in service use? Soc Sci Med. 2004 Jul;59(2):361-76.
241. Hunter DJ. Getting a grip on clinical variations in hospital services. BMJ. 2004 Mar 13;328(7440):610.
242. Ng AK, Li S, Neuberg D, Silver B, Weeks J, Mauch P. Factors influencing treatment recommendations in early-stage Hodgkin's disease: a survey of physicians. Ann Oncol. 2004 Feb;15(2):261-9.
243. Panella M, Marchisio S, Di Stanislao F. Reducing clinical variations with clinical pathways: do pathways work? Int J Qual Health Care. 2003 Dec;15(6):509-21.
244. [No authors listed]. Minimize practice variation by focusing on 'top priority conditions'. Perform Improv Advis. 2003 Sep;7(9):119-21, 117.
245. DeSalvo KB, Block JP, Muntner P, Merrill W. Predictors of variation in office visit interval assignment. Int J Qual Health Care. 2003 Oct;15(5):399-405.
246. Owen RR, Fischer EP, Kirchner JE, Thrush CR, Williams DK, Cuffel BJ, Elliott CE, Booth BM. Clinical practice variations in prescribing antipsychotics for patients with schizophrenia. Am J Med Qual. 2003 Jul-Aug;18(4):140-6.
247. de Jong JD, Groenewegen PP, Westert GP. Mutual influences of general practitioners in partnerships. Soc Sci Med. 2003 Oct;57(8):1515-24.
248. Krumholz HM, Chen J, Rathore SS, Wang Y, Radford MJ. Regional variation in the treatment and outcomes of myocardial infarction: investigating New England's advantage. Am Heart J. 2003 Aug;146(2):242-9.
249. Margenthaler JA, Meier JD, Virgo KS, Johnson DY, Goshima K, Chan D, Handler BS, Johnson FE. Geographic variation in posttreatment surveillance intensity for patients with cutaneous melanoma. Am J Surg. 2003 Aug;186(2):194-200.
250. Chung PJ, Chung J, Shah MN, Meltzer DO. How do residents learn? The development of practice styles in a residency program. Ambul Pediatr. 2003 Jul-Aug;3(4):166-72.
251. O'Hare AM, Dudley RA, Hynes DM, McCulloch CE, Navarro D, Colin P, Stroupe K, Rapp J, Johansen KL. Impact of surgeon and surgical center characteristics on choice of permanent vascular access. Kidney Int. 2003 Aug;64(2):681-9.
252. Stafford RS. Feedback intervention to reduce routine electrocardiogram use in primary care. Am Heart J. 2003 Jun;145(6):979-85.
253. Joines JD, Hertz-Picciotto I, Carey TS, Gesler W, Suchindran C. A spatial analysis of county-level variation in hospitalization rates for low back problems in North Carolina. Soc Sci Med. 2003 Jun;56(12):2541-53.
254. Peköz EA, Shwartz M, Iezzoni LI, Ash AS, Posner MA, Restuccia JD. Comparing the importance of disease rate versus practice style variations in explaining differences in small area hospitalization rates for two respiratory conditions. Stat Med. 2003 May 30;22(10):1775-86.
255. Grytten J, Sørensen R. Practice variation and physician-specific effects. J Health Econ. 2003 May;22(3):403-18.
256. Jin Y, Marrie TJ, Carriere KC, Predy G, Houston C, Ness K, Johnson DH. Variation in management of community-acquired pneumonia requiring admission to Alberta, Canada hospitals. Epidemiol Infect. 2003 Feb;130(1):41-51.
257. Greer AL, Goodwin JS, Freeman JL, Wu ZH. Bringing the patient back in. Guidelines, practice variations, and the social context of medical practice. Int J Technol Assess Health Care. 2002 Fall;18(4):747-61.
258. Fisher ES, Wennberg JE. Health care quality, geographic variations, and the challenge of supply-sensitive care. Perspect Biol Med. 2003 Winter;46(1):69-79. Review. PubMed PMID: 12582271.
259. Krein SL, Hofer TP, Kerr EA, Hayward RA. Whom should we profile? Examining diabetes care practice variation among primary care providers, provider groups, and health care facilities. Health Serv Res. 2002 Oct;37(5):1159-80.
260. Davis P, Gribben B, Lay-Yee R, Scott A. How much variation in clinical activity is there between general practitioners? A multi-level analysis of decision-making in primary care. J Health Serv Res Policy. 2002 Oct;7(4):202-8.
261. Garg PP, Landrum MB, Normand SL, Ayanian JZ, Hauptman PJ, Ryan TJ, McNeil BJ, Guadagnoli E. Understanding individual and small area variation in the underuse of coronary angiography following acute myocardial infarction. Med Care. 2002 Jul;40(7):614-26.
262. Long MJ. An explanatory model of medical practice variation: a physician resource demand perspective. J Eval Clin Pract. 2002 May;8(2):167-74.
263. Savino JS, Ley C, Boisvert D, Friedman A, Mathew J, Koch C, Starr N, Mangano CM, Herskowitz A, Browner WS, Mangano DT; Multicenter Study of Perioperative Ischemia (McSPI) Research Group; Ischemia Research and Education Foundation (IREF). Practice pattern variability for myocardial revascularization: impact on resource use across 24 centers. J Cardiothorac Vasc Anesth. 2002 Apr;16(2):149-56.
264. Smellie WS, Galloway MJ, Chinn D, Gedling P. Is clinical practice variability the major reason for differences in pathology requesting patterns in general practice? J Clin Pathol. 2002 Apr;55(4):312-4.
265. Coady SF. Influencing physician practice variation. The future of evidence-based medicine portends use of information that is cumulative and evolutionary, not static. Health Manag Technol. 2002 Feb;23(2):34-6.
266. Zink A, Listing J, Ziemer S, Zeidler H; German Collaborative Arthritis Centres. Practice variation in the treatment of rheumatoid arthritis among German rheumatologists. J Rheumatol. 2001 Oct;28(10):2201-8
267. Sicotte C, Béland F. The effect of medical work groups on hospital resource use. Health Serv Manage Res. 2001 Aug;14(3):165-80.
268. Majeed A, Moser K, Maxwell R. Age, sex and practice variations in the use of statins in general practice in England and Wales. J Public Health Med. 2000 Sep;22(3):275-9.
269. Hébert PC, Wells G, Martin C, Tweeddale M, Marshall J, Blajchman M, Pagliarello G, Sandham D, Schweitzer I I, Boisvert D, Calder L. Variation in red cell transfusion practice in the intensive care unit: a multicentre cohort study. Crit Care. 1999;3(2):57-63.
270. Trinh N, Ngo HH. Practice variations in the management of sinusitis. J Otolaryngol. 2000 Aug;29(4):211-7.
271. Davis P, Gribben B, Scott A, Lay-Yee R. The "supply hypothesis" and medical practice variation in primary care: testing economic and clinical models of inter-practitioner variation. Soc Sci Med. 2000 Feb;50(3):407-18.
272. Wright JG, Hawker GA, Bombardier C, Croxford R, Dittus RS, Freund DA, Coyte PC. Physician enthusiasm as an explanation for area variation in the utilization of knee replacement surgery. Med Care. 1999 Sep;37(9):946-56.
273. Westert GP, Groenewegen PP. Medical practice variations: changing the theoretical approach. Scand J Public Health. 1999 Sep;27(3):173-80.
274. Dieppe P, Basler HD, Chard J, Croft P, Dixon J, Hurley M, Lohmander S, Raspe H. Knee replacement surgery for osteoarthritis: effectiveness, practice variations, indications and possible determinants of utilization. Rheumatology (Oxford). 1999 Jan;38(1):73-83.
275. Franks P, Zwanziger J, Mooney C, Sorbero M. Variations in primary care physician referral rates. Health Serv Res. 1999 Apr;34(1 Pt 2):323-9.
276. Ghali WA, Ash AS, Hall RE, Moskowitz MA. Variation in hospital rates of intraaortic balloon pump use in coronary artery bypass operations. Ann Thorac Surg. 1999 Feb;67(2):441-5.
277. Alexander JA, Lee SY, Griffith JR, Mick SS, Lin X, Banaszak-Holl J. Do market-level hospital and physician resources affect small area variation in hospital use? Med Care Res Rev. 1999 Mar;56(1):94-117.
278. Desai P. Responding to the challenge of clinical practice variations. Br J Ophthalmol. 1998 Oct;82(10):1101-2.
279. Cooper GS, Chak A, Way LE, Hammar PJ, Harper DL, Rosenthal GE. Endoscopic practice for upper gastrointestinal hemorrhage: differences between major teaching and community-based hospitals. Gastrointest Endosc. 1998 Oct;48(4):348-53.
280. Freeborn DK, Shye D, Mullooly JP, Eraker S, Romeo J. Primary care physicians' use of lumbar spine imaging tests: effects of guidelines and practice pattern feedback. J Gen Intern Med. 1997 Oct;12(10):619-25.
281. van Miltenburg-van Zijl AJ, Bossuyt PM, Nette RW, Simoons ML, Taylor TR. Cardiologists' use of clinical information for management decisions for patients with unstable angina: a policy analysis. Med Decis Making. 1997 Jul-Sep;17(3):292-7.
282. Peterson S, Eriksson M, Tibblin G. Practice variation in Swedish primary care. Scand J Prim Health Care. 1997 Jun;15(2):68-75.
283. Wennberg D, Dickens J Jr, Soule D, Kellett M Jr, Malenka D, Robb J, Ryan T Jr, Bradley W, Vaitkus P, Hearne M, O'Connor G, Hillman R. The relationship between the supply of cardiac catheterization laboratories, cardiologists and the use of invasive cardiac procedures in northern New England. J Health Serv Res Policy. 1997 Apr;2(2):75-80.
284. van Miltenburg-van Zijl AJ, Simoons ML, Bossuyt PM, Taylor TR, Veerhoek MJ. Variation in the use of coronary angiography in patients with unstable angina is related to differences in patient population and availability of angiography facilities, without affecting prognosis. Eur Heart J. 1996 Dec;17(12):1828-35.
285. Wennberg JE. Practice variations and the challenge to leadership. Spine (Phila Pa 1976). 1996 Jun 15;21(12):1472-8.
286. Katz BP, Freund DA, Heck DA, Dittus RS, Paul JE, Wright J, Coyte P, Holleman E, Hawker G. Demographic variation in the rate of knee replacement: a multi-year analysis. Health Serv Res. 1996 Jun;31(2):125-40.
287. Stoevelaar HJ, van de Beek C, Casparie AF, Nijs HG, McDonnell J, Janknegt RA. [Variation in the diagnosis and treatment of benign prostatic hyperplasia in urological practice]. Ned Tijdschr Geneeskd. 1996 Apr 13;140(15):837-42.
288. Casparie AF. The ambiguous relationship between practice variation and appropriateness of care: an agenda for further research. Health Policy. 1996 Mar;35(3):247-65.
289. Malone ML, Bajwa TK, Battiola RJ, Fortsas M, Aman S, Solomon DJ, Goodwin JS. Variation among cardiologists in the utilization of right heart catheterization at time of coronary angiography. Cathet Cardiovasc Diagn. 1996 Feb;37(2):125-30.
290. Weiner JP, Starfield BH, Powe NR, Stuart ME, Steinwachs DM. Ambulatory care practice variation within a Medicaid program. Health Serv Res. 1996 Feb;30(6):751-70.
291. Geller SE, Burns LR, Brailer DJ. The impact of nonclinical factors on practice variations: the case of hysterectomies. Health Serv Res. 1996 Feb;30(6):729-50.
292. Wennberg JE. On the appropriateness of small-area analysis for cost containment. Health Aff (Millwood). 1996 Winter;15(4):164-7.
293. Kuhn EM, Hartz AJ, Baras M. Correlation of rates of coronary artery bypass surgery, angioplasty, and cardiac catheterization in 305 large communities for persons age 65 and older. Health Serv Res. 1995 Aug;30(3):425-36.
294. Hampton JR. Practice variations, appropriateness and decision analysis. QJM. 1995 Jun;88(6):365-7.
295. Carlisle DM, Valdez RB, Shapiro MF, Brook RH. Geographic variation in rates of selected surgical procedures within Los Angeles County. Health Serv Res. 1995 Apr;30(1):27-42.
296. Kay E, Nuttall N. Clinical decision making--an art or a science? Part III: To treat or not to treat? Br Dent J. 1995 Feb 25;178(4):153-5.
297. Grilli R, Repetto F. Variation in use of breast-conserving surgery in Lombardia, Italy. Int J Technol Assess Health Care. 1995 Fall;11(4):733-40.
298. Poses RM, Cebul RD, Wigton RS. You can lead a horse to water—improving physicians' knowledge of probabilities may not affect their decisions. Med Decis Making. 1995 Jan-Mar;15(1):65-75.
299. Volinn E, Diehr P, Ciol MA, Loeser JD. Why does geographic variation in health care practices matter? (And seven questions to ask in evaluating studies on geographic variation). Spine (Phila Pa 1976). 1994 Sep 15;19(18 Suppl):2092S-2100S.
300. Goodman DC, Fisher ES, Gittelsohn A, Chang CH, Fleming C. Why are children hospitalized? The role of non-clinical factors in pediatric hospitalizations. Pediatrics. 1994 Jun;93(6 Pt 1):896-902.
301. Davidson G. "Does inappropriate use explain small-area variations in the use of health care services?" A critique. Health Serv Res. 1993 Oct;28(4):389-400; discussion 401-18.
302. Poses RM, Wigton RS, Cebul RD, Centor RM, Collins M, Fleischli GJ. Practice variation in the management of pharyngitis: the importance of variability in patients' clinical characteristics and in physicians' responses to them. Med Decis Making. 1993 Oct-Dec;13(4):293-301.
303. Kristiansen IS, Mooney G. The general practitioner's use of time: is it influenced by the remuneration system? Soc Sci Med. 1993 Aug;37(3):393-9.
304. Steinberg EP. Variations research. The physician perspective. Med Care. 1993 May;31(5 Suppl):YS86-8.
305. Longo DR. Patient practice variation. A call for research. Med Care. 1993 May;31(5 Suppl):YS81-5.
306. Wennberg JE. Future directions for small area variations. Med Care. 1993 May;31(5 Suppl):YS75-80.
307. Chassin MR. Explaining geographic variations. The enthusiasm hypothesis. Med Care. 1993 May;31(5 Suppl):YS37-44.
308. [No authors listed]. Small-area variations: what are they and what do they mean? Health Services Research Group. CMAJ. 1992 Feb 15;146(4):467-70.
309. Henke CJ, Epstein WV. Practice variation in rheumatologists' encounters with their patients who have rheumatoid arthritis. Med Care. 1991 Aug;29(8):799-812.
310. Keller RB. Public data and private doctors: Maine tackles treatment variations. J State Gov. 1991 Jul-Sep;64(3):83-6.
311. Feinglass J, Martin GJ, Sen A. The financial effect of physician practice style on hospital resource use. Health Serv Res. 1991 Jun;26(2):183-205.
312. Renwick M. Variations in surgery rates: implications for quality. Aust Clin Rev. 1991;11(4):159-63.
313. Maryniuk GA. Practice variation: learned and socio-economic factors. Adv Dent Res. 1990 Jun;4:19-24. Review. PubMed PMID: 2206209.
314. Keller RB, Chapin AM, Soule DN. Informed inquiry into practice variations: the Maine Medical Assessment Foundation. Qual Assur Health Care. 1990;2(1):69-75.
315. McMahon LF Jr, Newbold R. Variation in resource use within diagnosis-related groups. The effect of severity of illness and physician practice. Med Care. 1986 May;24(5):388-97.
316. Smits HL. Medical practice variations revisited. Health Aff (Millwood). 1986 Fall;5(3):91-6. PubMed PMID: 3792991.
317. [No authors listed]. Practice variations in the provision of health care services. American Medical Association. Conn Med. 1985 Jul;49(7):457-8, 461-2. PubMed PMID: 4028711.
318. Hare RL. Practice variations: how much is too much? PROs, medical societies must play a role. Internist. 1985 Apr;26(4):9-10.
319. Wennberg JE. Practice variations: why all the fuss? Internist. 1985 Apr;26(4):6-8.
320. Schwartz JS. The role of professional medical societies in reducing practice variations. Health Aff (Millwood). 1984 Summer;3(2):90-101.
321. Wennberg JE. Dealing with medical practice variations: a proposal for action. Health Aff (Millwood). 1984 Summer;3(2):6-32.
322. [No authors listed]. Dealing with medical practice variations: a proposal for action. ReViews. Health Aff (Millwood). 1984 Summer;3(2):33-62.
323. Atsma F, Elwyn G, Westert G. Understanding unwarranted variation in clinical practice: a focus on network effects, reflective medicine and learning health systems. International Journal for Quality in Health Care, 2020, 32(4), 271–274.
324. Begin AS, Hidrue MK, Lehrhoff S, Lennes IT, Armstrong K, Weilburg JB, del Carmen MG, Wasfy JH. Association of self-reported primary care physician tolerance for uncertainty with variations in resource use and patient experience. JAMA Network Open. 2022;5(9):e2229521.
325. Bruce BR, Leask J, De Vries BS, Shepherd HL. Midwives' perspectives of intravenous fluid management and fluid balance documentation in labour: A qualitative reflexive thematic analysis study. J Adv Nurs. 2023;79:749–761.
326. Cardona M, Craig L, Jones M, Byambasuren O, Obucina M, Hattingh L, Clark J, Glasziou P, Hoffmann T. Guideline adherence as an indicator of the extent of antithrombotic overuse and underuse: a systematic review. Global Heart. 2022; 17(1): 55.
327. Coates D, Donnolley N, Foureurh M, Spear V. Amanda HenrycExploring unwarranted clinical variation: The attitudes of midwives and obstetric medical staff regarding induction of labour and planned caesarean section. Women and Birth 2021; 34: 352–361.
328. de Graaff MR, Klaase JM, de Kleine R, Elfrink AKE, Swijnenburg RJ et al. Practice variation and outcomes of minimally invasive minor liver resections in patients with colorectal liver metastases: a population‑based study. Surgical Endoscopy (2023) 37:5916–5930.
329. de Man Y, Groenewoud S, Oosterveld-Vlug MG, Brom L, Onwuteaka-Philipsen BD, Westert GP, Atsma F. Regional variation in hospital care at the end-of-life of Dutch patients with lung cancer exists and is not correlated with primary and long-term care. International Journal for Quality in Health Care, 2020, 32(3), 190–195.
330. Ferrari A, Giannini A, Seghieri C, et al. Regional practice variation in pelvic organ prolapse surgery in Tuscany, Italy: a retrospective cohort study on administrative health data. BMJ Open 2023;13:e068145.
331. Frank‑Tewaag J, Bleek J, Günster C, Schneider U, Horenkamp‑Sonntag D, Marschall U, Franke S, Schlößler K, Donner‑Banzhoff N, Sundmacher L. Regional variation in coronary angiography rates: the association with supply factors and the role of indication: a spatial analysis. BMC Cardiovascular Disorders (2022) 22:72.
332. Frehn JL, Brewster A, Shortell SM, Rodriguez HP. comparing health care system and physician practice influences on social risk screening. Health Care Manage Rev. 2022 ; 47(1): E1–E10.
333. George LS, Epstein RM, Akincigil A, Saraiya B, Trevino KM, Kuziemski A, Pushparaj L, Policano E, Prigerson HG, Godwin K, Duberstein P. Psychological Determinants of Physician Variation in End-of-Life Treatment Intensity: A Systematic Review and Meta-Synthesis. J Gen Intern Med 38(6):1516–25.
334. Grove A, Pope C, Currie G, Clarke A. Paragons, Mavericks and Innovators. A typology of orthopaedic surgeons' professional identities. A comparative case study of evidence-based practice. Sociol Health Illn. 2022;44:59–80.
335. Halpern DJ, Clark-Randall A, Woodall J, Anderson J, Shah K. Reducing imaging utilization in primary care through implementation of a peer comparison dashboard. J Gen Intern Med 36(1):108–13.
336. Harris AHS, Meerwijk EL, Kamal RN, Sears ED, Curtin CM, Hawn M, Eisenberg D, Finlay AK, Hagedorn H, Marshall N, Mudumbai SC. Variation in surgeons’ requests for general anesthesia when scheduling carpal tunnel release. Hand 2020; 15(5): 608–614.
337. Hek K, van Esch TEM, Lambooij A, Weesie YM, van Dijk L. Guideline adherence in antibiotic prescribing to patients with respiratory diseases in primary care: prevalence and practice variation. Antibiotics 2020, 9, 571; doi:10.3390/antibiotics9090571.
338. Hodgson T, Burton-Jones A, Donovan R, Sullivan C. The Role of Electronic Medical Records in Reducing Unwarranted Clinical Variation in Acute Health Care: Systematic Review JMIR Med Inform 2021;9(11):e30432.
339. Kalkman GA, Kramers C, van Dongen RT, Schers HJ, van Boekel RLM, Bos JM, et al. Practice variation in opioid prescribing for noncancer pain in Dutch primary care: A retrospective database study. PLoS ONE 2023; 18(2): e0282222.
340. Kollerup A, Wadmann S, Bek T, Kjellberg J. National clinical guidelines and treatment centralization do not guarantee consistency in healthcare delivery. A mixed-methods study of wet age-related macular degeneration treatment in Denmark. Health policy 126 (2022) 1291–1302.
341. Lecarpentier P, Gandré C, Coldefy M, Ellini A, Trichard C. Use of electroconvulsive therapy for individuals receiving inpatient psychiatric care on a nationwide scale in France: Variations linked to health care supply. Brain Stimulation 2022;15: 201e210
342. Larkins NG, Lim W, Goh C, Francis A, McCarthy H, Kim S, Wong G, Craig JC. Timing of kidney replacement therapy among children and young adults. CJASN 2023; 18: 1041–1050.
343. Leonard LD, de Araujo TB, Quinn C, Thomas MB, Beaty L, Mott NM, Colborn K, Heelan AA, Tevis SEA, Christian N, Arhendt G, Gleisner AL. De‑implementation of axillary dissection in women undergoing mastectomy for breast cancer. Ann Surg Oncol. 2023; 30:5692–5702.
344. Luijten J, Vissers P, Brom L, de Bièvre M, Buijsen J, Rozema T, Haj Mohammad N, van Duijvendijk P, Kouwenhoven E, Eshuis W, Rosman C, Siersema P, van Laarhoven H, Verhoeven R, Nieuwenhuijzen G, Westerman M. Clinical variation in the organization of clinical pathways in esophagogastric cancer, a mixed method multiple case study. BMC Health Services Research. 2022; 22:527.
345. MacKay EJ, Zhang B, Shah RM, Augoustides JG, Groeneveld PW, Desai ND. Predictors of intraoperative echocardiography: Analysis of The Society of Thoracic Surgeons database. Ann Thorac Surg 2023;115:1289-96.
346. Manderbacka K, Satokangas M, Arffman M, Reissell E, Keskimäki I, Leyland AH. Explaining regional variation in elective hip and knee arthroplasties in Finland 2010 − 2017—a register‑based cohort study. BMC Health Services Research. 2022; 22:891.
347. Mansoor N, Gulati S, Fredriksli OA, Salvesen O, Solheim O. Epidemiology and practice variations of shunt surgery for hydrocephalus: a nationwide registry–based study. J Neurosurg. 2023; 139:892–900.
348. Moore Z, Aynge G, Carr C, Horton A, Jones H, Murphy N, Payne M, McCarthy C, Murdoch JM. A Clinical Support App for routine wound management: reducing practice variation, improving clinician confidence and increasing formulary compliance. Int Wound J. 2022;19:1263–1275.
349. Morrow A, Steinberg J, Chan P, Tiernan G, Kennedy E, Egoroff N, Hilton D, Sankey L, Venchiarutti R, Hayward A, Pearn A, McKay S, Debono D, Hogden E, Taylor N. In person and virtual process mapping experiences to capture and explore variability in clinical practice: application to genetic referral pathways across seven Australian hospital networks. Translational Behavioral Medicine. 2023; 13: 561–570.
350. Müskens J, van Dulmen S, Wiersma T, Burgers J, Hek K, Westert G, Kool R. Low-value pharmaceutical care among Dutch GPs: a retrospective cohort study. Br J Gen Pract 2022; DOI: <https://doi.org/10.3399/BJGP.2021.0625>.
351. Naranjo D, Doll J, Maynard C, Beaver K, Bansal A, Helfrich C. Practice pattern variation in adoption of new and evolving percutaneous coronary intervention procedures. Journal of Interventional Cardiology. 2023; Article ID 2488045.
352. Offerhaus P, van Haaren-Ten Haken TM, Keulen JKJ, de Jong JD, Brabers AEM, Verhoeven CJM, Scheepers H, Nieuwenhuijze M. Regional practice variation in induction of labor in the Netherlands: Does it matter? A multilevel analysis of the association between induction rates and perinatal and maternal outcomes. PLoS ONE 2023;18(6): e0286863.
353. Shashar S, Ellen M, Codish S, Davidson E, Novack V. Medical practice variation among primary care physicians: 1 decade, 14 health services, and 3,238,498 patient-years. Ann Fam Med 2021;19:30-37.
354. Sakowski JA, Song PH. The extent hospital organizational factors influence inpatient care delivery: a case study looking at knee and hip replacement surgery. Health Services Insights. 2022; 15: 1–9.
355. Tohidi M, Mann SM, Groome PA. Total hip arthroplasty versus hemiarthroplasty for treatment of femoral neck fractures: a population-based analysis of practice variation in Ontario, Canada. Bone Joint J 2023;105-B(2):180–189.
356. Tohidi M, Mann SM, Groome PA. Total hip arthroplasty for displaced femoral neck fracture: Survey of orthopaedic surgeons in Ontario, Canada. Injury 54 (2023) 1740–1747.
357. Upasani V, Burns J, Bastrom T, Baldwin K, Schoenecker J, Shore B. Practice variation in the surgical management of children with acute hematogenous osteomyelitis. J Pediatr Orthop 2022;42:e520–e525.
358. Van Munster J, de Weerdt V, Halperin I, Zamanipoor Najafabadi A, van Benthem P, Schoonman G, Moojen W, van den Hout W, Atsma F, Peul W. Practice variation research in degenerative lumbar disc surgery: a literature review on design characteristics and outcomes. Global Spine Journal 2022, Vol. 12(8) 1841–1851.
359. Wayne M, Valley T, Arenberg D, De Cardenas J, Prescott H. Temporal trends and variation in bronchoscopy use for acute respiratory failure in the United States. Chest. 2023; 163(1):128-138.
360. Wertli MM, Schlapbach JM, Haynes AG, Scheuter C, Jegerlehner SN, Panczak R, Chiolero A, Rodondi N, Aujesky D. Regional variation in hip and knee arthroplasty rates in Switzerland: A populationbased small area analysis. PLoS ONE. 2020; 15(9): e0238287.
361. Wolf M, Hasselström JK, Carlsson A, Von Euler M, Hasselström J. Identifying factors explaining practice variation in secondary stroke prevention in primary care: a cohort study based on all patients with ischaemic stroke in the Stockholm region. BMJ Open 2022;12:e064277.
362. Wu B, David G. Information, relative skill, and technology abandonment. Journal of Health Economics. 2022; 83:102596.
363. Zuckerman J, Coburn N, Callum J, Mahar AL, Lin Y, Turgeon AF, McLeod R, Pearsall E, Martel G, Hallet J. Evaluating variation in perioperative red blood cell transfusion for patients undergoing elective gastrointestinal cancer surgery. Surgery. 2023; 173: 392e400.

APPENDIX C: PRISMA-ScR CHECKLIST

**Preferred Reporting Items for Systematic reviews and Meta-Analyses extension for Scoping Reviews (PRISMA-ScR) Checklist**

| **SECTION** | **ITEM** | **PRISMA-ScR CHECKLIST ITEM** | **REPORTED ON PAGE #** |
| --- | --- | --- | --- |
| **TITLE** | | | |
| Title | 1 | Identify the report as a scoping review. | Page 1 / title page |
| **ABSTRACT** | | | |
| Structured summary | 2 | Provide a structured summary that includes (as applicable): background, objectives, eligibility criteria, sources of evidence, charting methods, results, and conclusions that relate to the review questions and objectives. | Page 2 |
| **INTRODUCTION** | | | |
| Rationale | 3 | Describe the rationale for the review in the context of what is already known. Explain why the review questions/objectives lend themselves to a scoping review approach. | Page 3 and 4 |
| Objectives | 4 | Provide an explicit statement of the questions and objectives being addressed with reference to their key elements (e.g., population or participants, concepts, and context) or other relevant key elements used to conceptualize the review questions and/or objectives. | Page 5 |
| **METHODS** | | | |
| Protocol and registration | 5 | Indicate whether a review protocol exists; state if and where it can be accessed (e.g., a Web address); and if available, provide registration information, including the registration number. | Not applicable, no published protocol |
| Eligibility criteria | 6 | Specify characteristics of the sources of evidence used as eligibility criteria (e.g., years considered, language, and publication status), and provide a rationale. | Table 1, page 6 and 7 |
| Information sources* | 7 | Describe all information sources in the search (e.g., databases with dates of coverage and contact with authors to identify additional sources), as well as the date the most recent search was executed. | Page 6 and 7 |
| Search | 8 | Present the full electronic search strategy for at least 1 database, including any limits used, such that it could be repeated. | Appendix A |
| Selection of sources of evidence† | 9 | State the process for selecting sources of evidence (i.e., screening and eligibility) included in the scoping review. | Page 6 and 7 |
| Data charting process‡ | 10 | Describe the methods of charting data from the included sources of evidence (e.g., calibrated forms or forms that have been tested by the team before their use, and whether data charting was done independently or in duplicate) and any processes for obtaining and confirming data from investigators. | Page 7 and 8 |
| Data items | 11 | List and define all variables for which data were sought and any assumptions and simplifications made. | Page 8 |
| Critical appraisal of individual sources of evidence§ | 12 | If done, provide a rationale for conducting a critical appraisal of included sources of evidence; describe the methods used and how this information was used in any data synthesis (if appropriate). | Page 8 and 9 |
| Synthesis of results | 13 | Describe the methods of handling and summarizing the data that were charted. | Page 8 and 9 |
| **RESULTS** | | | |
| Selection of sources of evidence | 14 | Give numbers of sources of evidence screened, assessed for eligibility, and included in the review, with reasons for exclusions at each stage, ideally using a flow diagram. | Figure 2 |
| Characteristics of sources of evidence | 15 | For each source of evidence, present characteristics for which data were charted and provide the citations. | Table 2, Appendix B |
| Critical appraisal within sources of evidence | 16 | If done, present data on critical appraisal of included sources of evidence (see item 12). | Not applicable; done at a more general level |
| Results of individual sources of evidence | 17 | For each included source of evidence, present the relevant data that were charted that relate to the review questions and objectives. | Table 3 and 4 summarise the relevant data |
| Synthesis of results | 18 | Summarize and/or present the charting results as they relate to the review questions and objectives. | Page 9 and 10 |
| **DISCUSSION** | | | |
| Summary of evidence | 19 | Summarize the main results (including an overview of concepts, themes, and types of evidence available), link to the review questions and objectives, and consider the relevance to key groups. | Page 9 and 10 |
| Limitations | 20 | Discuss the limitations of the scoping review process. | Page 11 and 13 |
| Conclusions | 21 | Provide a general interpretation of the results with respect to the review questions and objectives, as well as potential implications and/or next steps. | Page 11 - 13 |
| **FUNDING** | | | |
| Funding | 22 | Describe sources of funding for the included sources of evidence, as well as sources of funding for the scoping review. Describe the role of the funders of the scoping review. | Page 1 / title page |

JBI = Joanna Briggs Institute; PRISMA-ScR = Preferred Reporting Items for Systematic reviews and Meta-Analyses extension for Scoping Reviews.

* Where *sources of evidence* (see second footnote) are compiled from, such as bibliographic databases, social media platforms, and Web sites.

† A more inclusive/heterogeneous term used to account for the different types of evidence or data sources (e.g., quantitative and/or qualitative research, expert opinion, and policy documents) that may be eligible in a scoping review as opposed to only studies. This is not to be confused with *information sources* (see first footnote).

‡ The frameworks by Arksey and O’Malley (6) and Levac and colleagues (7) and the JBI guidance (4, 5) refer to the process of data extraction in a scoping review as data charting*.*

§ The process of systematically examining research evidence to assess its validity, results, and relevance before using it to inform a decision. This term is used for items 12 and 19 instead of "risk of bias" (which is more applicable to systematic reviews of interventions) to include and acknowledge the various sources of evidence that may be used in a scoping review (e.g., quantitative and/or qualitative research, expert opinion, and policy document).

*From:* Tricco AC, Lillie E, Zarin W, O'Brien KK, Colquhoun H, Levac D, et al. PRISMA Extension for Scoping Reviews (PRISMAScR): Checklist and Explanation. Ann Intern Med. 2018;169:467–473. [doi: 10.7326/M18-0850](http://annals.org/aim/fullarticle/2700389/prisma-extension-scoping-reviews-prisma-scr-checklist-explanation).
